# Design, synthesis, and molecular dynamics simulation studies of some novel kojic acid fused 2-amino-3-cyano-4*H*-pyran derivatives as tyrosinase inhibitors

**DOI:** 10.1186/s13065-024-01134-1

**Published:** 2024-02-22

**Authors:** Zahra Najafi, Maryam Zandi Haramabadi, Gholamabbas Chehardoli, Ahmad Ebadi‬, Aida Iraji

**Affiliations:** 1 Department of Medicinal Chemistry, School of Pharmacy, Hamadan University of Medical Sciences, Hamadan, Iran; 2 Department of Medicinal Chemistry, School of Pharmacy, Medicinal Plants and Natural Products Research Center, Hamadan University of Medical Sciences, Hamadan, Iran; 3 Stem Cells Technology Research Center, Shiraz University of Medical Sciences, Shiraz, Iran; 4 Research Center for Traditional Medicine and History of Medicine, Department of Persian Medicine, School of Medicine, Shiraz University of Medical Sciences, Shiraz, Iran

**Keywords:** Kojic acid, Benzyloxy benzylidene, 2-Amino-3-cyano-4*H*-pyran, Molecular dynamics simulation, Tyrosinase inhibitors

## Abstract

A novel series of kojic acid fused 2-amino-3-cyano-4H-pyran derivatives were synthesized via a multicomponent reaction involving kojic acid, benzyloxy benzaldehyde, and malonitrile as tyrosinase inhibitors. Subsequently, the structures of the compounds were characterized using FT-IR, ^1^H-, and ^13^C-NMR spectroscopic analyses. The designed compounds fall into three series: (1) 4-benzyloxy-phenyl kojopyran **6a**–**e**, (2) 3-benzyloxy- phenyl kojopyran derivatives **6f**–**j**, and (3) 4-benzyloxy-3-methoxy-phenyl kojopyran derivative **6 k**–**o**. The assessment of tyrosinase inhibition activity was conducted using L-Dopa as the substrate. Among synthesized compounds, 2-amino-4-(4-((4-fluorobenzyl)oxy)phenyl)-6-(hydroxymethyl)-8-oxo-4,8-dihydropyrano[3,2-*b*]pyran-3-carbonitrile (**6b**) demonstrated the highest antityrosinase activity with a competitive inhibition pattern (IC_50_ = 7.69 ± 1.99 μM) as compared to the control agent kojic acid (IC_50_ = 23.64 ± 2.56 µM). Since compound **6b** was synthesized as a racemic mixture, in silico studies were performed for both *R* and *S* enantiomers. The *R*- enantiomer showed critical interactions compared with the *S*-enantiomer. Specifically, it established hydrogen bonds and hydrophobic interactions with crucial and highly conserved amino acids within the enzyme's binding site in the target protein. Moreover, the molecular dynamics simulations revealed that compound **6b** demonstrated significant interactions with essential residues of the binding site, resulting in a stable complex throughout the entire simulation run. The drug-like and ADMET properties predictions showed an acceptable profile for compound **6b**. Thus, it can serve as a drug candidate to develop more potent antityrosinase agents due to its low toxicity and its high inhibition activity.

**Supplementary Information:**

The online version contains supplementary material available at 10.1186/s13065-024-01134-1.

## Introduction

Melanogenesis is a physiological process that produces a natural pigment called melanin which plays an important role in preventing sun-induced skin injuries in all organisms [1]. Modulating melanogenesis is a widely employed approach for addressing irregular skin pigmentation using both pharmaceutical and cosmetic interventions [2].

Tyrosinase enzyme plays a key role in the early stage of melanogenesis by catalyzing the oxidation of phenol to o-quinone observed. Enzyme activity may cause disturbances in pigment production [3]. Excessive production and hyperpigmentation of melanin are seen in various skin disorders such as freckles, age spots, pregnancy spots, melisma, pigmented scars caused by acne, and melanoma as one of the deadly types of cancer [4].

Browning in plants and fruits such as mushrooms, apples, pears, and bananas results from overactivity of the tyrosinase enzyme. It leads to a decrease in these products' quality and commercial value [5]. On the other hand, the defense mechanisms, such as skin shedding and wound healing in insects, are accomplished by tyrosinase enzyme. As a result, the inhibition of tyrosinase enzyme in plants and insects can have great importance in agriculture industries [6].

Tyrosinase is also related to neurodegenerative diseases such as Parkinson’s by oxidizing excess dopamine to produce dopamine quinone. These highly reactive species induce neural damage and cell death, and the production of a pigment called neuromelanin in the human brain and some neurons. An imbalance in dopamine quinones and neuromelanin levels is associated with the development of neurodegenerative disorders [7, 8].

Kojic acid isolated from the fungus *Aspergillus oryzae* is a well-known competitive inhibitor against antityrosinase and free radical scavenger agent [9]. As a result, the development of new kojic acid Although using products containing kojic acid may be considered safe for most people, there are some risks and possible side effects such as sunburn and contact dermatitis in some people, especially those with sensitive skin. As a result, the development of new kojic acid-related compounds that have fewer side effects is considered by many scientists [10, 11]. On another hand, pyran-fused compounds have long captivated the attention of synthetic and biological researchers due to their diverse range of chemical and biological properties [12].

**Fig. 1:**
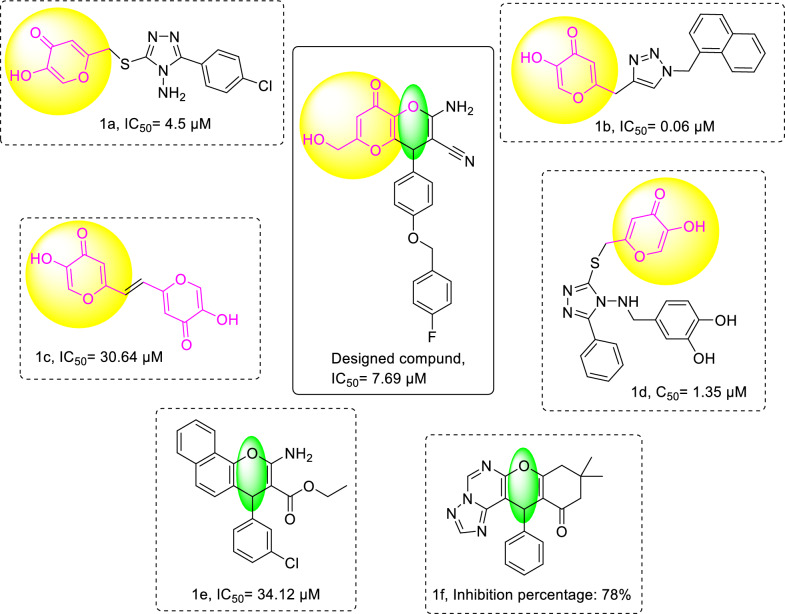
Kojic acid and 4H-pyran related structures as tyrosinase inhibitors and newly designed hybrid

Therefore, medicinal chemists have used kojic acid and pyran core (compounds** a**, **b**, **d**, **c**, Fig. 1) as effective pharmacophores in designing the new tyrosinase inhibitors [12–17]. Based on the biological importance of kojic acid compounds and in continuation of our research programs [18, 19], some novel kojic acid fused 2-amino-3-cyano-4*H*-pyran derivatives were designed, synthesized, and evaluated as tyrosinase inhibitors. Afterward, kinetic and in silico studies including ADMET, molecular docking, and molecular dynamics simulation, were conducted to predict the potential of potent derivatives as ideal drug candidates for further studies.

## Results and discussion

### Chemistry

**Fig. 2:**
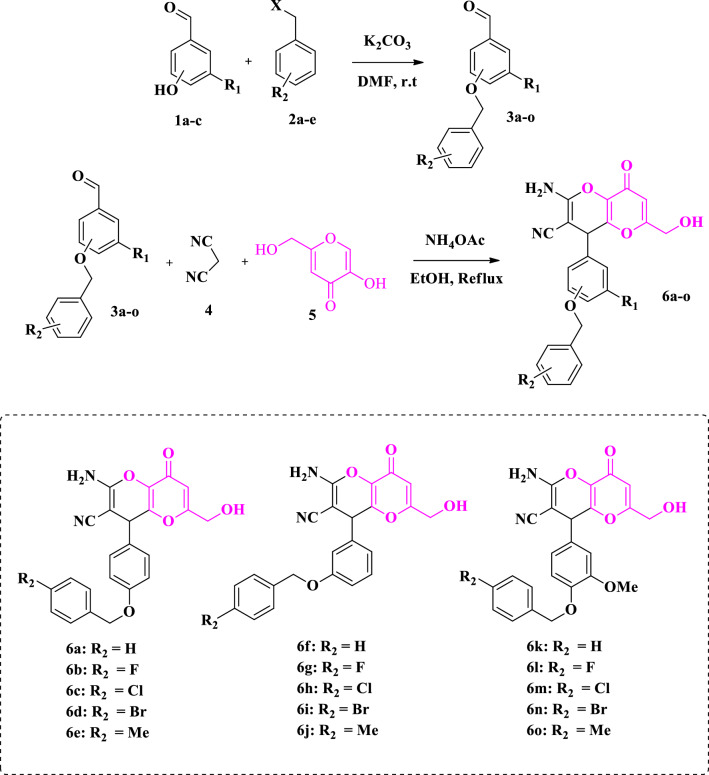
General procedure for the synthesis of kojic acid fused 2-amino-3-cyano-4*H*-pyran derivatives **6a**–**o**

The synthetic routes for the synthesis of kojic acid fused 2-amino-3-cyano-4*H*-pyran derivatives **6a–o** were shown in Fig. 2. For this purpose, in the first step, various benzyloxy-benzaldehydes **3a–o** were obtained by the nucleophilic reaction between 4-hydroxyl-benzaldehydes or 3-hydroxyl-benzaldehydes or vanillin (**1a**–**c**) and benzyl halide derivatives **2a–e** in the presence of potassium carbonate in DMF at the room temperature [20]. In the second step, the multicomponent reaction between benzyloxy-benzaldehydes **3a**–**o**, malonitrile **4** and kojic acid **5** was conducted in ethanol (5 ml) in reflux condition to produce the target compounds **6a**–**o** in good to acceptable yields [21]. The structure of all new kojic acid derivatives was confirmed by ^1^H, ^13^C NMR, IR spectroscopy, and elemental analysis.

### Tyrosinase inhibitory activity

The inhibitory activities of the kojic acid fused 2-amino-3-cyano-4*H*-pyran derivatives **6a-o** on mushroom tyrosinase were investigated using kojic acid as a positive control by evaluation of their potential to inhibit enzymatic oxidation of L-Dopa. All results are presented as mean ± SE in Table 1. The synthesized products shown in Fig. 2 can be divided into three series based on starting aldehyde: (1) 4-benzyloxy-phenyl kojopyran **6a**–**e** (4-hydroxy-benzaldehyde derived compounds), (2) 3-benzyloxy- phenyl kojopyran derivatives **6f**–**j** (3-hydroxy-benzaldehyde derived compounds), and (3) 4-benzyloxy-3-methoxy-phenyl kojopyran derivative **6 k**–**o** (vanillin derived compounds).

**Table 1:** Tyrosinase inhibitory activities of compounds **6a-o**

Entry	Compounds	R_1_	R_2_	% Inhibition at 80 µM^a^	% Inhibition at 30 µM^a^	IC_50_ (µM)
1	6a	H	H	47.90 ± 3.90	–	–
2	6b	H	4-F	89.27 ± 2.59	79.81 ± 6.18	7.69 ± 1.99
3	6c	H	4-Cl	50.01 ± 4.34	44.09 ± 2.94	79.25 ± 3.30
4	6d	H	4-Br	66.36 ± 3.64	48.86 ± 3.53	33.96 ± 2.53
5	6e	H	4-Me	48.63 ± 6.37	–	–
6	6f	H	H	42.27 ± 2.72	–	–
7	6g	H	4-F	88.63 ± 6.36	77.95 ± 4.54	9.72 ± 2.47
8	6h	H	4-Cl	67.95 ± 4.54	65.90 ± 5.09	10.66 ± 4.96
9	6i	H	4-Br	67.72 ± 7.27	59.54 ± 5.44	15.34 ± 3.17
10	6j	H	4-Me	20.45 ± 4.54	–	–
11	6k	OMe	H	10.25 ± 3.48	–	–
12	6l	OMe	4-F	49.09 ± 4.91	–	–
13	6m	OMe	4-Cl	49.31 ± 6.12	–	–
14	6n	OMe	4-Br	21.36 ± 3.63	–	–
15	6o	OMe	4-Me	44.31 ± 4.18	–	–
16	Kojic acid	–	–	–	–	23.64 ± 2.56

^a^Values represent means ± SD of three independent experiments

Among synthesized compounds **6a**–**o**, compound **6b** exhibited the most inhibitory activity against tyrosinase enzyme with an IC_50_ value of 7.69 ± 1.99 µM, which was better than kojic acid inhibitory activity (IC_50_ = 23.64 ± 2.56 µM). As seen in Table 1, the first and second groups, compounds **6a**–**j**, indicated better inhibitory activities than the third group **6k**–**o**. It seems that the methoxy group and the phenyl ring diminished antityrosinase activity. Both electronic and spatial effects can reduce the third group's activity, although spatial effects play a more important role. In the first series, compound **6a**, without substituting the benzyl moiety, was inactive against the tyrosinase enzyme. The introduction of electron-withdrawing groups (EWGs), including 4-F, 4-Cl, and 4-Br on the benzyl pendant group were, generated compounds **6b**, **6c**, and **6d** with IC_50_ values of 7.69, 79.25, and 33.96 µM, respectively. Notably, electron-withdrawing effect orders are F > Cl > Br. The compound **6b** with fluorine at benzyl moiety's 4th position illustrated better inhibitory activity than chlorine and bromine atoms. Also, a comparison between compound **6c** having 4-chlorine and **6e** having 4-methyl as electron donating group (EDG) at the benzyl moiety with the same size showed that electron-withdrawing groups could produce more potent agents than the electron donating group. The second series of the synthesized compounds **6f**–**j** were structurally different from the first series since pendant benzyl moiety connected to phenyl at the 3rd position. The compound **6f** exhibited no inhibitory activity towards tyrosinase.

Similar to the first series introduction of EWGs, including 4-F, 4-Cl, and 4-Br led to improve antityrosinase activities in the compounds **6g**, **6h**, and **6i** with IC_50_ values of 9.72, 10.66, and 15.34 µM. It also introduced the 4-methyl group as an EDG at the benzyl pendant moiety that created compound **6j** without any activity. Fluorine as a small EWG apparently produced more effective ligand-enzyme interaction than chlorine and bromine atoms as large EWGs.

### Kinetic studies

**Fig. 3:**
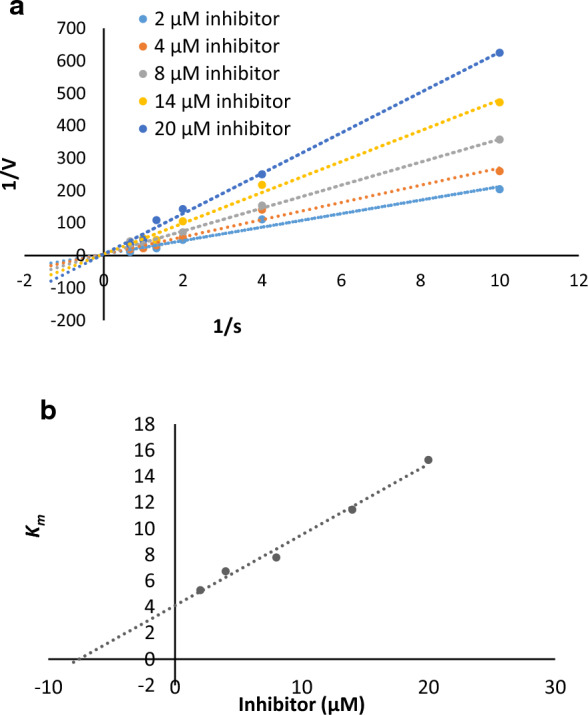
**a** Lineweaver − Burk plot of tyrosinase inhibition by different concentrations of **6b** in the presence of L-DOPA. **b** The secondary Lineweaver–Burk plot of compound **6b**

Mechanism and type of inhibition were elucidated through kinetic investigations. Lineweaver utilized Burk plot analysis to determine the enzyme inhibition mode, employing the most potent derivative **6b** (see Fig. 3A). Lineweaver–Burk plots, depicting *1/V* against 1/[S], were constructed to illustrate the inhibition of tyrosinase by **6b** across varying concentrations of both **6b** and L-Dopa, the substrate. In this context, *K*_*m*_ represents the Michaelis–Menten constant, while *V*_*max*_ signifies the maximum reaction velocity.

Upon analyzing the data, it was observed that the enzyme's *V*_*max*_ remained consistent, while the *K*_*m*_ values exhibited a rise when subjected to increasing concentrations of compound **6b** (depicted in Fig. 3A). Based on these findings, it can be inferred that the inhibitory effect of compound **6b** is competitive. This deduction is further supported by the secondary graph (Fig. 3B) portraying the relationship between Km and varied concentrations of compound **6b**, facilitating the estimation of inhibition constants (*K*_*i*_ = 7.57 µM).

### Drug-likeness prediction

To remove molecules with unfavorable properties from screening libraries and increase the chance of drug discovery, the drug-likeness concept was first proposed by Lipinski known as the rule of five to define the physicochemical ranges of an oral drug. This rule includes the molecular weight (MW ≤ 500), the number of hydrogen bonding acceptors (HBA ≤ 10), the number of hydrogen bonding acceptors (HBD ≤ 5), the lipophilicity index (logP ≤ 5), rotatable bond count (RBC ≤ 10) and polar surface area (PSA ≤ 140) of compounds. Two online servers, including pkCSM and SwissADME, were used to calculate these parameters [22, 23]. Some compounds (**6b**, **6d**, **6g**, **6h,** and **6i**) with the best anti-tyrosinase activities were selected to evaluate physicochemical properties. Data in Table 2 confirm that all selected compounds successfully passed Lipinski's rule.

**Table 2:** Physicochemical properties of the selected synthesized compounds (6b, 6d, 6g, 6h, and 6i)

Compd.	MW	HBA	HBD	Log P	RBC	TPSA
6b	420.39	7	2	2.61	5	118.71
6d	481.30	6	2	2.95	5	118.71
6g	420.39	7	2	2.61	5	118.71
6h	436.84	6	2	2.74	5	118.71
6i	481.30	6	2	2.95	5	118.71

### Prediction of ADMET properties

ADMET describes the Absorption, Distribution, Metabolism, Excretion, and Toxicity of a pharmaceutical compound within an organism. ADMET properties enable drug developers to investigate the safety and efficacy of drugs for preclinical and clinical development. Table 3 shows ADMET prediction for the selected compounds (**6b**, **6d**, **6g**, **6h**, and **6i**) calculated by pkCSM and SwissADME online servers [22, 23]. The estimated Human Intestinal Absorption (HIA) of the selected compounds is high, which illustrates high absorption by gastrointestinal tracts. All compounds are predicted to have acceptable skin permeability. The steady-state volume of distribution (VDss) refers to the volume where the total medication content is distributed to attain a consistent concentration in the blood plasma. The acceptable value for VDss is 0.45 > LogVDss > − 0.15. All synthesized compounds are within the defined range. Regarding metabolism, all compounds were predicted not to be inhibitors or substrates of CYP450 2D6 while expected to be CYP450 3A4 and 2C9 inhibitors. For the excretion, none of the compounds is expected to be substrates for the Renal Organic Cation Transporter 2 (OCT2). Finally, all products are not considered toxic regarding AMES, skin sensitization and hERG I channel inhibition. Also, the calculated skin permeability for all compounds was sited in the allowed range. According to these data, it could be predicted that the selected potent compounds (**6b**, **6d**, **6g**, **6h**, and **6i**) can have an acceptable pharmacokinetic profile.

**Table 3:** ADMET^a^ prediction of the synthesized compounds 6b, 6d, 6g, 6h, and 6i

Compd	Absorption		Distribution ^b^	Metabolism ^b^				Excretion ^b^	Toxicity^b^		
	HIA%	Skin permeability (logKp)	VDss (log L/Kg)	CYP3A4 inhibition	CYP2C9 inhibition	CYP2C19 inhibition	CYP2D6 inhibition	Renal OCT2 substrate	AMES toxicity	Skin Sensitization	hERG1 inhibitor
**6b**	89.92	− 2.74	0.127	Yes	Yes	No	No	No	No	No	No
**6d**	87.38	− 2.75	0.224	Yes	Yes	Yes	No	Yes	No	No	No
**6g**	92.78	− 2.74	0.076	Yes	Yes	No	No	No	No	No	No
**6h**	90.64	− 2.75	–	–	–	–	–	–	–	–	–
**6i**	90.38	− 2.75	–	–	–	–	–	–	–	–	–

^a^HIA (Human Intestinal Absorption): > 80% is high and < 30% is poor; VDss (steady-state volume of distribution): log L/Kg: > 0.45 is high and < − 0.15 is low. A compound is considered to have low skin permeability if it has a logKp > − 2.5. PkCSM and SwissADME online servers calculated all data. ^b^The software could not predict parameters for these compounds (−)

### Molecular docking study

**Fig. 4:**
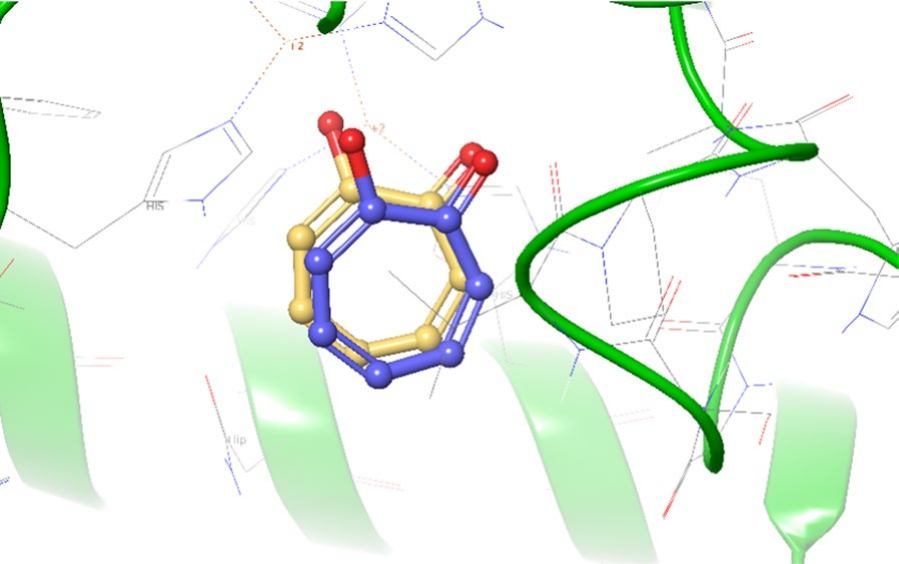
The superimpose structure of crystalographic tropolone (yellow) vs docked tropolone (blue)

This study employed the induced fit docking method to analyze the most potent compound **6b** binding mode within the tyrosinase active site. To ensure the reliability of the docking protocol, re-docking experiment was performed with the crystalographic inhibitor tropolone into the enzyme’s active site. The resulting superimposed structures of the docked and crystallized tropolone showed good agreement, with a record RMSD value of less than 2 Å (Fig. 4).

Next, induced fit docking was applied to **6b** as the most potent analog. It should be noted that compound **6b** was produced as a racemic mixture including *R-* and *S-* enantiomers; as a result, comprehensive molecular docking studies were undertaken to evaluate both enantiomers in the active site of tyrosinase.

**Fig. 5:**
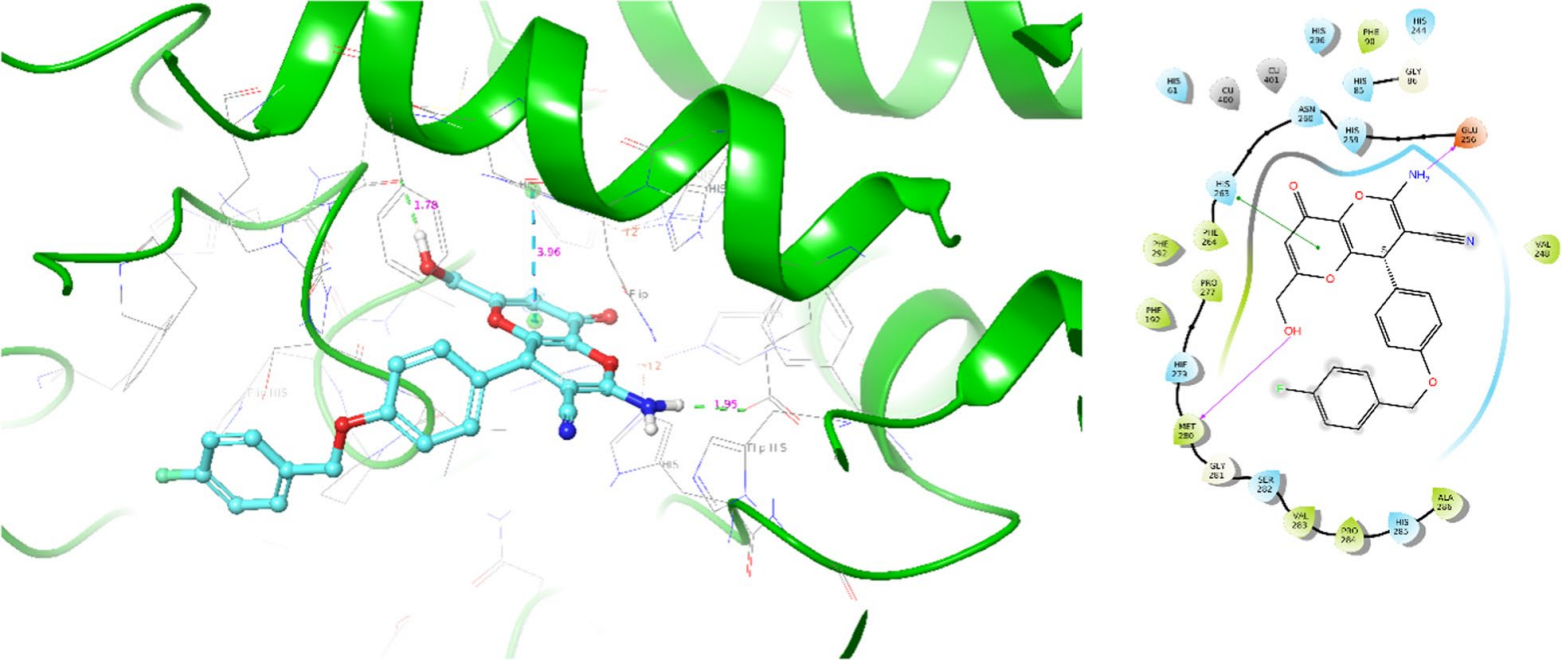
3D and 2D interaction of *S*-enantiomer of compound **6b** in complex with tyrosinase

The results of *S*- enantiomer of compound **6b** within the tyrosinase binding site are depicted in Fig. 5, which recorded the binding energy of − 6.304 kcal/mol. The OH group of *S*- enantiomer **6b** exhibited H-bound interaction with Met280 (1.76 Å), and the other H-bound interaction is recorded between NH_2_ and Glu256 residue (1.95 Å). Kojopyran moiety also participated in pi-pi stacking interaction with His263 (3.96 Å).

**Fig. 6:**
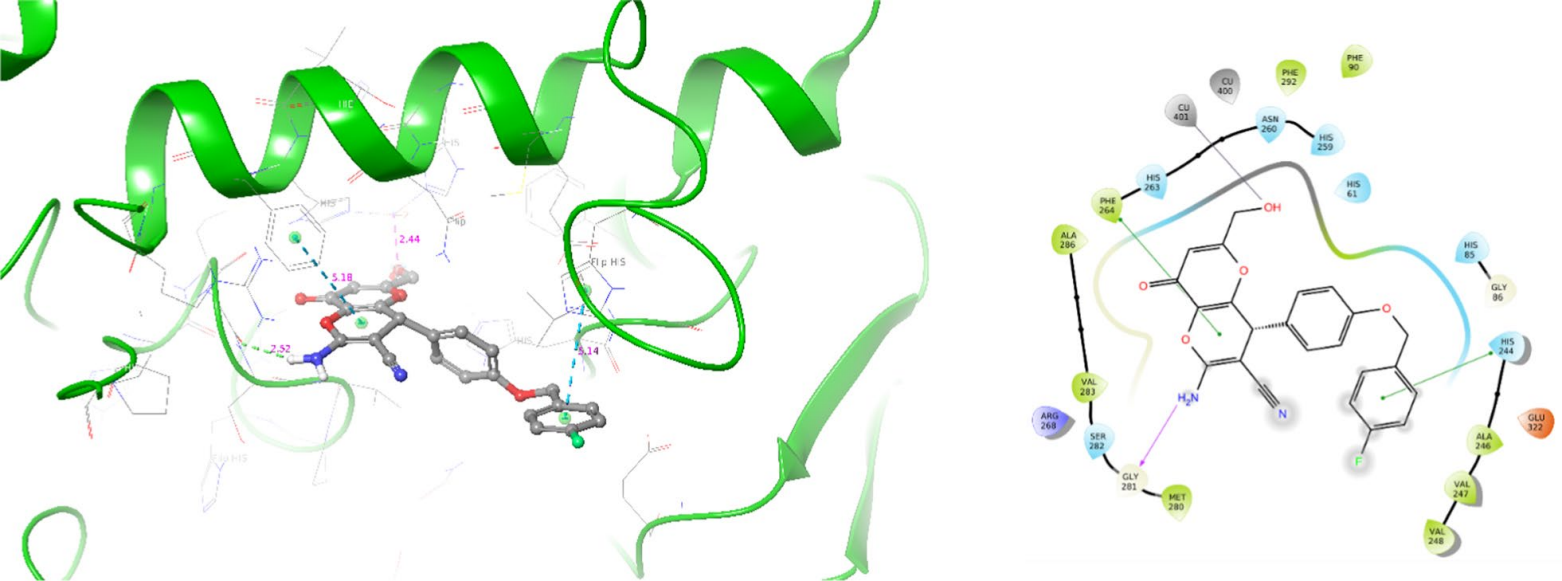
3D and 2D interaction of *R*-enantiomer of compound **6b** in complex with tyrosinase

The interaction between tyrosinase and *R*- enantiomer of compound **6b** is depicted in Fig. 6 with the binding energy of − 6.825 kcal/mol. In this complex, the kojopyran moiety engaged in a hydrogen bonding interaction with Gly281 (2.52 Å). In contrast, the NH group of kojopyran participated in a pi-pi stacking interaction with Phe264 (5.18 Å). Furthermore, the OH group of *R*- enantiomer **6b** exhibited metal chelation with Cu401 (2.44 Å) involving residues categorized as critical amino acids for the binding. Additionally, the 4-fluoro-benzyl terminal of the compound formed another important pi-pi stacking interaction with His244 (5.14 Å), further contributing to the compound's binding affinity.

### Molecular dynamics simulations

It is worth noting that the molecular docking study revealed no significant disparities in the binding energies between the *S*- and *R*- enantiomers. Consequently, molecular dynamics simulations were accompanied to extract the optimal enantiomeric conformation with better interaction and stability with the enzyme.

**Fig. 7:**
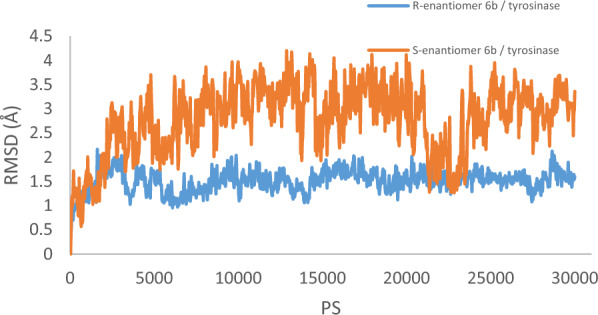
RMSD of the tyrosinase backbone in complexed *R*- enantiomer **6b** (in blue) *S*- enantiomer **6b** (in orange) for over 30 ns molecular dynamics simulation time

An analysis of the root mean square deviation (RMSD) data throughout the molecular dynamics run indicated that during the initial three nanoseconds (ns)* R*-enantiomer **6b**, there was a significant increase in RMSD, reaching a value of 2 Å (Fig. 7). However, after this initial phase, a gradual decrease in RMSD occurred, indicating a convergence towards a more stable configuration. Subsequently, the RMSD values recorded steady fluctuations around 1.5 Å. This behavior suggested that the simulation had reached equilibrium, and the protein–ligand complex remained relatively stable during the molecular dynamics run. Such stability is crucial for potent enzyme inhibitor, as it demonstrates a consistent and prolonged inhibition of the enzyme activity. In contrast, *S*-**6b** exhibited less stability than the *R*- **6b** isomer with an average value of 3.5 Å.

**Fig. 8:**
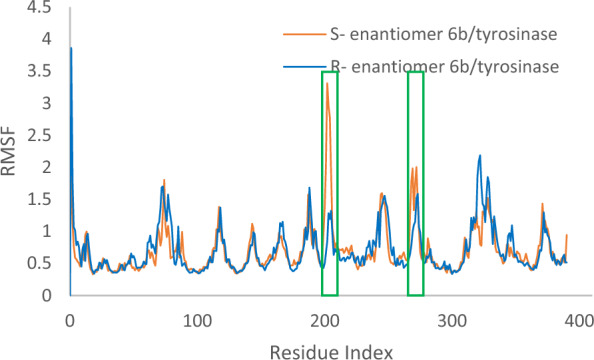
**a** RMSF of the *R*- enantiomer **6b** (in blue) *S*- enantiomer **6b** (in orange) for over 30 ns molecular dynamics simulation time

The root mean square fluctuation (RMSF) plot of *R*-**6b** and *S*-**6b**, shown in Fig. 8, illustrates the flexibility of the enzyme structure concerning its secondary structure elements. It can be observed that the N- and C-terminal ends of the enzyme and the unstructured regions exhibited higher fluctuations compared to the secondary structure elements. This is common as terminal regions and unstructured parts are generally more dynamic. The results revealed that a reduction in residue motion was observed upon binding the ligand to tyrosinase. This reduction can be attributed to the non-bonding interactions between the ligand and the enzyme. The most significant disparities in RMSF between the two systems were found at residue indices that directly interacted with the ligand. These residues experienced reduced fluctuations, indicating that the ligand binding stabilized these regions by restricting their motion Overall, the RMSF analysis demonstrated that the enzyme- **6b** (*R*) complex exhibited lower RMSF values than the apoenzyme. More stability was further corroborated by the RMSF analysis of *R*-**6b** (highlighted in green), which demonstrated fewer fluctuations in the ligand’s atoms *vs* of *S*- **6b**. This suggests an increased propensity for interactions with the enzyme’s binding site.

**Fig. 9:**
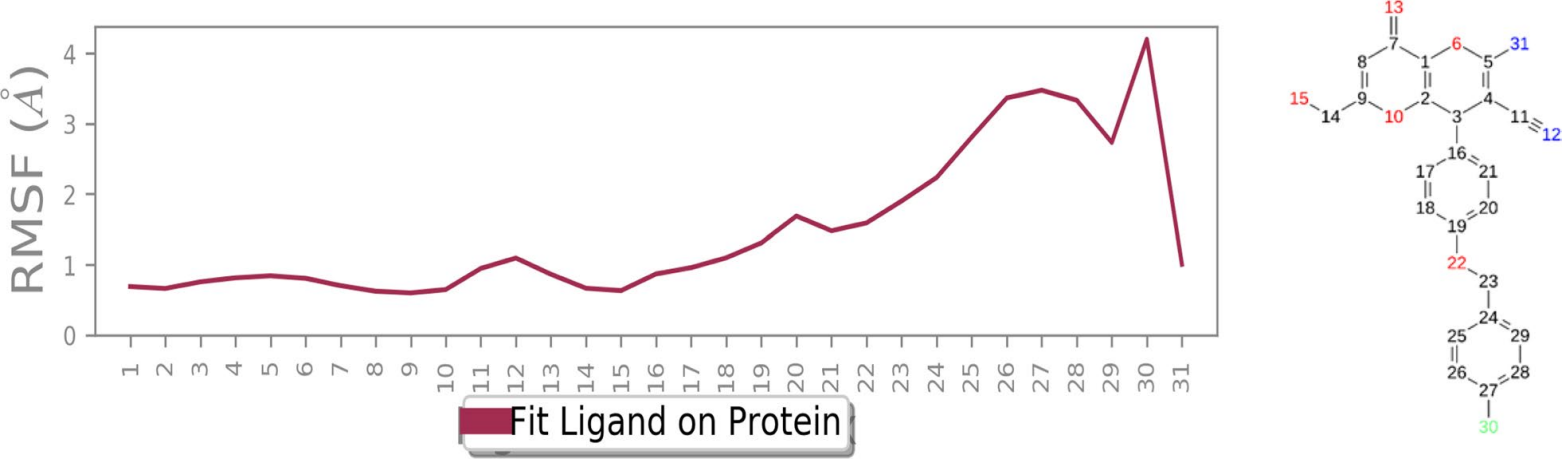
RMSF graph of the heavy atoms of *R*- enantiomer **6b** in complex with tyrosinase. The structure of *R*- enantiomer **6b** is depicted, exhibiting the regions of the molecule with the highest fluctuations

**Fig. 10:**
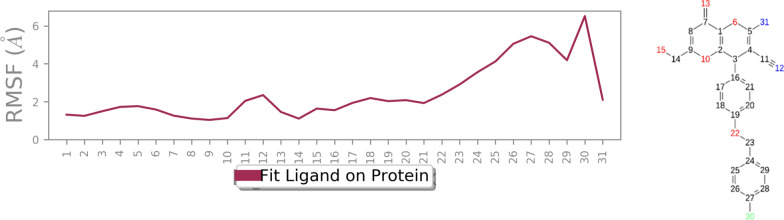
RMSF graph of the heavy atoms of *S*- enantiomer **6b** in complex with tyrosinase. The structure of *S*- enantiomer **6b** is depicted, exhibiting the regions of the molecule with the highest fluctuations

Figure 9 shows RMSF values for the heavy atoms of ligand *R*-**6b** bound to tyrosinase. All *R*- enantiomer **6b** atoms, except those in the 4-fluoro benzyl substituted regions, exhibit RMSF values below 2 Å. This low fluctuation indicates that these atoms form a stable complex with tyrosinase due to strong intermolecular interactions, limiting their movements during the molecular dynamics simulation. However, RMSF values for the heavy atoms of ligand *S*-**6b** (Fig. 10) demonstrated higher RMSF value, particularly evidenced by the RMSD value exceeding 4 Å for the *para*-fluorobenzyl moiety. These results suggest that ligand *R*-**6b** has the potential to be an effective inhibitor and more stable in the tyrosinase active site.

**Fig. 11:**
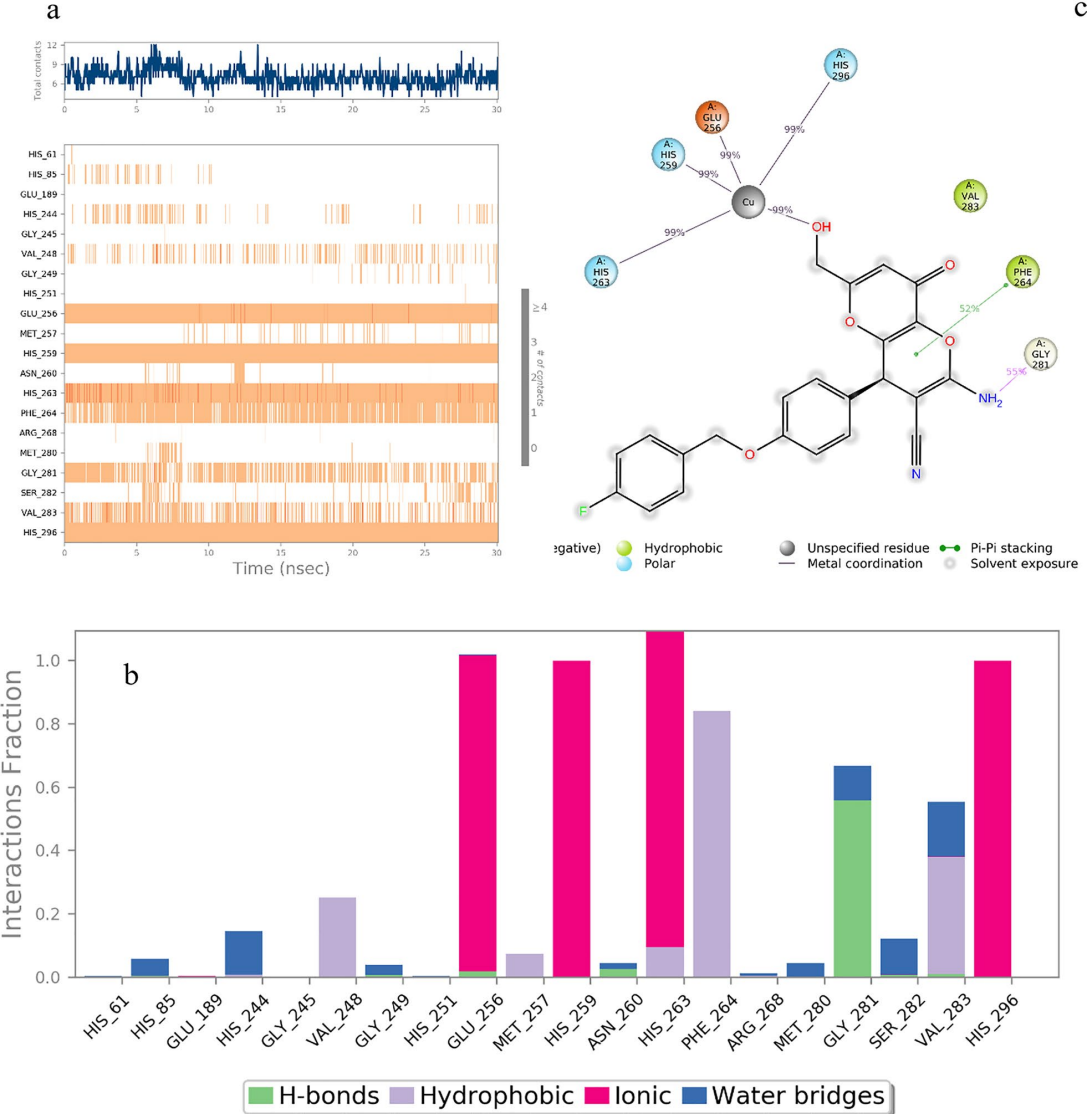
**a** A timeline representation of the interactions and contacts, **b** Protein-* R*- enantiomer **6b** contacts based on the type of interactions, **c** Protein residues interactions with *R*- enantiomer atom

Figure 11a presents a detailed timeline of interactions during the molecular dynamics simulation. The enzyme’s interactions with the ligand were continuously monitored throughout the simulation, and these interactions have been categorized and summarized in Fig. 8b. The interactions of *R*- enantiomer **6b** with the active site pocket of the enzyme were observed to occur for more than 50% of the simulation duration. These interactions can be summarized as follows (Fig. 8c):

The OH group of the kojopyran ring effectively participated in a critical metal chelation interaction with the Cu cofactor, persisting throughout 99% of the molecular dynamics run. This metal chelation interaction also affected the interactions with other amino acids, including His256, His263, His296, and Glu256.

The NH group of kojopyran demonstrated hydrogen bonding interaction with Gly281, which occurred approximately 55% of the simulation time.

Additionally, the mentioned ring structure of the ligand was involved in a pi-pi stacking interaction with Phe264. Interestingly, there is a high similarity between the docking interaction of *R*- enantiomer **6b** and the results of molecular dynamics. A similar interaction is observed in molecular dynamics simulation *vs* molecular docking, so the OH group of the kojopyran ring participated in a critical metal chelation interaction with the Cu cofactor, and NH of the kojopyran ring showed hydrogen bonding interaction with Gly281. Similarly, phe264 exhibited pi-pi stacking interaction with kojopyran.

**Fig. 12:**
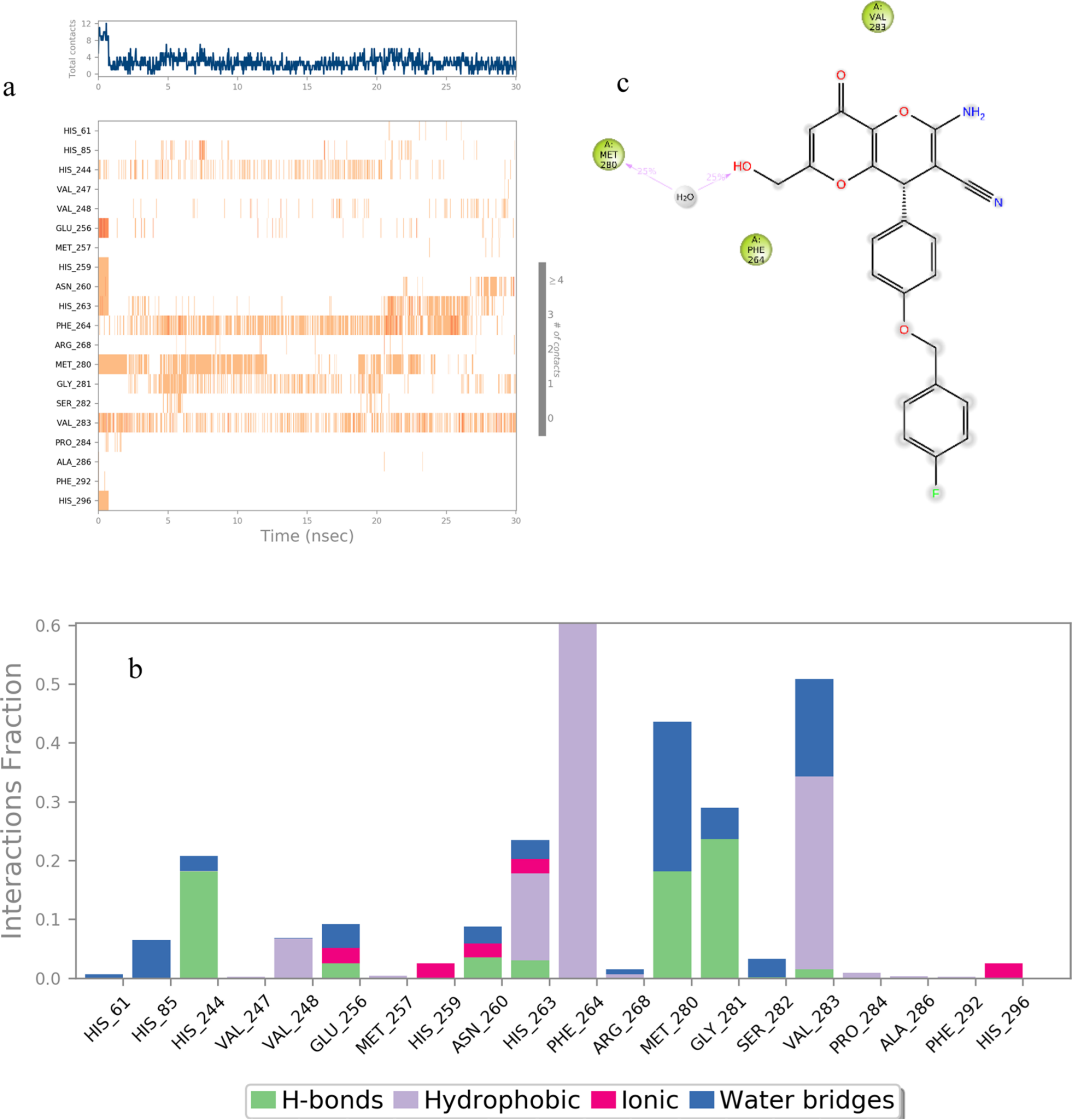
**a** A timeline representation of the interactions and contacts, **b** Protein-* S*- enantiomer of **6b** contacts based on the type of interactions, **c** Protein residues interactions with *S*- enantiomer of **6b** atom

The timeline of interactions during the molecular dynamics simulation of *S*- enantiomer of **6b** is demonstrated in Fig. 12a. Also, the enzyme's interactions with the ligand were continuously monitored throughout the simulation, summarized in Fig. 12b. Importantly, the *S*- enantiomer displayed no interactions with the enzyme's binding site throughout the simulation in 30% run time, and just one interaction with Met290 mediated with water is presented in 25% of simulation run (12c). The interaction with this residue was also recorded in molecular docking study.

Consequently, molecular dynamics evaluations of *R* and* S*- enantiomer of **6b** provided valuable insights into the binding interactions between **6b** enantiomers and the tyrosinase active site, supporting the role of *R*- enantiomer of **6b** as a promising inhibitor.

## Conclusions

As a result, a new series of some novel kojic acid fused 2-amino-3-cyano-4*H*-pyran derivatives as tyrosinase inhibitors were designed, synthesized, and asse* R*- enantiomer assessed against tyrosinase enzyme. Among synthesized derivatives, compound **6b** showed the most potent antityrosinase effect with an IC_50_ value of 7.69 ± 1.99 μM and a competitive mode of kinetic inhibition as compared to kojic acid as the control agent (23.64 ± 2.56 µM).

The in silico study *R*- enantiomer of compound **6b** within the enzyme’s binding site demonstrated significant interactions with critical and highly conserved amino acids, including hydrogen bonds and hydrophobic interactions. Additionally, molecular dynamics simulations of compound **6b** (*R*) revealed strong and stable interactions of the kojopyran moiety with essential residues and the Cu cofactor in the binding site. Furthermore, during the simulation run, the tyrosinase-**6b** complex was stable. The drug-like and ADMET properties predictions showed an acceptable profile for these agents. Our results proposed that compound **6b** can serve as a drug candidate to develop more potent antityrosinase agents.

## Experimental Section

### Chemistry

All chemical reagents were sourced from Merck and Sigma and employed without additional purification steps. Melting points were determined using a Stuart melting point smp3 apparatus. The structural integrity of all synthesized compounds was validated by utilizing IR spectra, ^1^H-NMR, ^13^C-NMR, and MS spectroscopy. The NMR data (^1^H and ^13^C) and IR spectra were acquired using a Bruker 400-NMR and ALPHA FT-IR spectrometer on KBr disks. Chemical shifts (*δ*) and coupling constants (*J*) were expressed in parts per million (ppm) and Hertz, respectively. The atom numbering of the target compounds, derived from their IUPAC nomenclature, was employed for the assignment of ^1^H-NMR data. The original spectra of the investigated compounds are provided as Additional file 1.

#### *General procedure for the synthesis of Benzyloxy-benzaldehyde derivatives* 3a-o

In a 25 ml round-bottom flask, a mixture of 3-hydroxybenzaldehyde or 4-hydroxybenzaldehyde or vanillin **1a**–**c** (1 mmol), various benzyl halides **2a**–**e** (1.1 mmol), and potassium carbonate (1.1 mmol) in dimethylformamide solvent 5 ml (DMF) were stirred at room temperature until the reaction was complete. The progress of the reaction was monitored using thin-layer chromatography (TLC). After the completion of reaction, ice was added to the mixture and stirred for 10 min. Then, the precipitate **3a**–**o** were filtered and washed with cold water. They were used for the next step without further purification [20].

#### General procedure for the synthesis of dihydropyrano [3, 2-b] pyran-3-carbonitrile 6a-o

In a 25 ml round-bottom flask, a three-component reaction including benzyloxy aldehyde derivatives **3a**–**o** (1.2 mmol), kojic acid **4** (1 mmol), and malononitrile **5** (1.2 mmol) was refluxed in the presence of ammonium acetate as the catalyst in ethanol. TLC was used to follow the reaction progress. Following the completion of the reaction, the products **6a**–**o** were isolated through filtration and subjected to a wash with an ethanol/water mixture [21].

2-Amino-4-(4-(benzyloxy)phenyl)-6-(hydroxymethyl)-8-oxo-4,8-dihydropyrano[3,2-*b*]pyran-3-carbonitrile (6a).

White solid; Mp: 221–224 °C; IR (KBr): υ (cm^−1^) = 3400, 3327, 3207, 2874, 2199, 1677. ^1^H NMR (400 MHz, DMSO-*d*_6_) δ (ppm): 4.10 (m, 2H, -CH_2_), 4.74 (s, 1H, -CH), 5.09 (s, 2H, -CH_2_), 5.70 (m, 1H, -CH_2_), 6.63 (s, 1H, -OH), 7.03 (d, 2H, -NH_2_), 7.20 (dd, *J* = 8.8 Hz, 2H, Aromatic), 7.36 (m, 5H, Aromatic).^13^C NMR (101 MHz, DMSO-*d*_*6*_) δ (ppm): 165.6, 168.2, 159.1, 157.9, 149.2, 136.9, 136.1, 133.0, 128.9, 128.4, 127.9, 127.7, 119.3, 115.0, 111.3, 69.2, 59.1 and 55.9. Anal.Calcd for C_23_H_18_N_2_O_5_: C, 68.65; H, 4.51; N, 6.96. Found: C, 68.43; H, 4.75; N, 6.90.

2-Amino-4-(4-((4-fluorobenzyl)oxy)phenyl)-6-(hydroxymethyl)-8-oxo-4,8-dihydropyrano[3,2-*b*]pyran-3-carbonitrile (6b).

White solid; Mp: 227–230 °C; IR (KBr): υ (cm^−1^) = 3271, 2207, 1645, 1598. ^1^H NMR (400 MHz, DMSO-*d*_6_) δ (ppm): 2.32 (s, 3H, -CH_3_), 4.11 (m, 2H, -CH_2_), 4.72 (s, 1H, -CH), 5.08 (s, 2H, -CH_2_), 5.70 (t, 1H, -CH_2_), 6.35 (s, 1H, -OH), 6.87 (d, 2H, -NH_2_), 6.89 (m, 1H, Aromatic), 7.00 (m, 4H, Aromatic), 7.24 (m, 1H, Aromatic), 7.54 (m, 2H, Aromatic).^13^C NMR (101 MHz, DMSO-*d*_*6*_) δ (ppm): 169.5, 168.1, 162.9, 160.5, 159.3, 158.6, 148.8, 142.3, 136.4, 133.1, 120.1, 119. 2, 115.3, 114.4, 113.8, 111.4, 68.5, 59.1 and 55.5. Anal.Calcd for C_23_H_17_FN_2_O_5_: C, 65.71; H, 4.08; N, 4.52. Found: C, 65.82; H, 4.15; N, 4.61.

2-Amino-4-(4-((4-chlorobenzyl)oxy)phenyl)-6-(hydroxymethyl)-8-oxo-4,8- dihydropyrano[3,2-*b*]pyran-3-carbonitrile (6c).

White solid; Mp: 236–239 °C; IR (KBr): υ (cm^−1^) = 3175, 2197, 1638, 1509. ^1^H NMR (400 MHz, DMSO-*d*_6_) δ (ppm): 4.10 (m, 2H, -CH_2_), 4.74 (s, 1H, -CH), 5.09 (s, 2H, -CH_2_), 5.70 (m, 1H, -CH_2_), 6.33 (s, 1H, -OH), 7.01 (d, 2H, -NH_2_), 7.22 (m, 4H, Aromatic), 7.48 (td, 4H, Aromatic).^13^C NMR (101 MHz, DMSO-*d*_*6*_) δ (ppm): 169.5, 168.1, 159.1, 157.8, 149.2, 136.4, 136.1, 133.0, 132.5, 130.1, 129.5, 128.9, 120.9, 119.3, 115.9, 111.3, 68.4, 59.1 and 55.8. Anal.Calcd for C_23_H_17_ClN_2_O_5_: C, 65.71; H, 4.08; N, 4.52. Found: C, 65.82; H, 4.15; N, 4.61.

2-Amino-4-(4-((4-bromobenzyl)oxy)phenyl)-6-(hydroxymethyl)-8-oxo-4,8 dihydropyrano[3,2-*b*]pyran-3-carbonitrile (6d).

White solid; Mp: 214–216 °C; IR (KBr): υ (cm^−1^) = 3406, 3273, 2191, 1636, 1488. ^1^H NMR (400 MHz, DMSO-*d*_6_) δ (ppm): 4.10 (m, 2H, -CH_2_), 5.08 (s, 1H, -CH), 5.09 (s, 2H, -CH_2_), 5.70 (m, 1H, -CH_2_), 6.33 (s, 1H, -OH), 7.02 (d, 2H, -NH_2_), 7.22 (m, 4H, Aromatic), 7.41 (td, 2H, Aromatic), 7.59 (t, 2H, Aromatic) .^13^C NMR (101 MHz, DMSO-*d*_*6*_) δ (ppm): 169.5, 168.1, 160.4, 159.1, 157.8, 149.2, 136.4, 136.1, 133.2, 131.5, 130.1, 129.8, 128.9, 120.9, 119.3, 115.9, 111.3, 68.4, 59.1 and 55.8. Anal.Calcd for C_23_H_17_BrN_2_O_5_: C, 57.40; H, 3.56; N, 5.82. Found: C, 57.58; H, 3.45; N, 5.71.

2-Amino-6-(hydroxymethyl)-4-(4-((4-methylbenzyl)oxy)phenyl)-8-oxo-4,8-dihydropyrano[3,2-*b*]pyran-3-carbonitrile (6e).

Yellow solid; Mp: 215–218 °C; IR (KBr): υ (cm^−1^) = 3417, 2193, 1635, 1512.^1^H NMR (400 MHz, DMSO-*d*_6_) δ (ppm): 2.32 (s, 3H, -CH_3_), 4.10 (m, 2H, -CH_2_), 4.73 (s, 1H, -CH), 5.04 (s, 2H, -CH_2_), 5.70 (m, 1H, -CH_2_), 6.33 (s, 1H, -OH), 7.01 (d, 2H, -NH_2_), 7.19 (m, 6H, Aromatic), 7.35 (m, 2H, Aromatic).^13^C NMR (101 MHz, DMSO-*d*_*6*_) δ (ppm): 169.5, 168.1, 159.1, 157.9, 149.2, 137.1, 136.1, 133.9, 132.9, 129.1, 128.9, 127.8, 119.3, 115.9, 115.0, 111.3, 69.1, 59.1, 55.9 and 20.8. Anal.Calcd for C_24_H_20_N_2_O_5_: C, 69.22; H, 4.84; N, 6.73. Found: C, 69.28; H, 4.93; N, 6.65.

2-Amino-4-(3-(benzyloxy)phenyl)-6-(hydroxymethyl)-8-oxo-4,8-dihydropyrano[3,2-b]pyran-3-carbonitrile (6f).

White solid; Mp: 235–238 °C; IR (KBr): υ (cm^−1^) = 3271, 2191, 1637, 1487.

^1^H NMR (400 MHz, DMSO-*d*_6_) δ (ppm): 4.10 (m, 2H, -CH_2_), 4.24 (s, 1H, -CH), 5.10 (s, 2H, -CH_2_), 5.69 (t, 1H, -CH_2_), 6.86 (s, 1H, -OH), 6.86 (m, 1H, Aromatic), 6.98 (s, 1H, Aromatic), 7.31 (d, 2H, -NH_2_), 7.34 (m, 4H, Aromatic), 7.45 (m, 2H, Aromatic).^13^C NMR (101 MHz, DMSO-*d*_*6*_) δ (ppm): 169.5, 168.2, 159.2, 158.7, 142.4, 136.8, 136.4, 130.1, 128.4, 127.9, 127.8, 120.1, 119.2, 114.4, 113.8, 111.4, 69.2, 59.1 and 55.5. Anal.Calcd for C_23_H_18_N_2_O_5_: C, 68.65; H, 4.51; N, 6.96. Found: C, 68.77; H, 4.39; N, 6.88.

2-Amino-4-(3-((4-fluorobenzyl)oxy)phenyl)-6-(hydroxymethyl)-8-oxo-4,8-dihydropyrano[3,2-b]pyran-3-carbonitrile (6g).

White solid; Mp: 239–242 °C; IR (KBr): υ (cm^−1^) = 3192, 2194, 1634, 1508. ^1^H NMR (400 MHz, DMSO-*d*_6_) δ (ppm): 4.10 (m, 2H, -CH_2_), 4.75 (s, 1H, -CH), 5.08 (s, 2H, -CH_2_), 5.69 (m, 1H, -CH_2_), 6.33 (s, 1H, -OH), 7.02 (d, 2H, Aromatic), 7.22 ( m, 6H, Aromatic), 7.52 (d, 2H, -NH_2_). ^13^C NMR (101 MHz, DMSO-*d*_*6*_) δ (ppm): 169.6, 168.1, 162.9, 160.5 159.1, 157.9, 149.2, 136.1, 133.2, 130.1, 129.9, 128.9, 119.3, 115.3, 111.3, 68.5, 59.1 and 55.8. Anal.Calcd for C_23_H_17_FN_2_O_5_: C, 65.71; H, 4.08; N, 6.66. Found: C, 65.67; H, 4.15; N, 6.16.

2-Amino-4-(3-((4-chlorobenzyl)oxy)phenyl)-6-(hydroxymethyl)-8-oxo-4,8-dihydropyrano[3,2-b]pyran-3-carbonitrile (6h).

White solid; Mp: 232–236 °C; IR (KBr): υ (cm^−1^) = 3406, 3273, 2191, 1636. ^1^H NMR (400 MHz, DMSO-*d*_6_) δ (ppm): 4.10 (m, 2H, -CH_2_), 4.77 (s, 1H, -CH), 5.10 (s, 2H, -CH_2_), 5.70 (m, 1H, -CH_2_), 6.89 (s, 1H, -OH), 6.97 (d, 2H, Aromatic), 6.99 (d, 1H, Aromatic), 7.30 (d, 2H, -NH_2_), 7.31 (m, 1H, Aromatic), 7.33 (m, 4H, Aromatic). ^13^C NMR (101 MHz, DMSO-*d*_*6*_) δ (ppm): 169.5, 168.1, 159.2, 158.5, 148.8, 142.5, 136.4, 135.9, 132.4, 130.1, 129.6, 128.4, 120.2, 119.2, 114.4, 113.8, 111.4, 68.3, 59.1, and 55.5. Anal.Calcd for C_23_H_17_ClN_2_O_5_: C, 63.24; H, 3.92; N, 6.41. Found: C, 63.17; H, 4.05; N, 6.38.

2-Amino-4-(3-((4-bromobenzyl)oxy)phenyl)-6-(hydroxymethyl)-8-oxo-4,8-dihydropyrano[3,2-b]pyran-3-carbonitrile (6i).

White solid; Mp: 236–238 °C; IR (KBr): υ (cm^−1^) = 3302, 2193, 1639, 1510. ^1^H NMR (400 MHz, DMSO-*d*_6_) δ (ppm): 4.10 (m, 2H, -CH_2_), 4.77 (s, 1H, -CH), 5.09 (s, 2H, -CH_2_), 5.69 (m, 1H, -CH_2_), 6.34 (s, 1H, -OH), 6.87 (d, 2H, Aromatic), 6.91 (d, 1H, Aromatic), 7.26 (d, 2H, -NH_2_), 6.99 (m, 1H, Aromatic), 7.35 (dd, 2H, Aromatic), 7.60 (m, 2H, Aromatic). ^13^C NMR (101 MHz, DMSO-*d*_*6*_) δ (ppm): 169.5, 168.1, 159.2, 158.5, 148.8, 142.5, 136.4, 135.9, 132.4, 130.1, 129.6, 128.4, 120.2, 119.2, 114.4, 113.8, 111.4, 68.3, 59.1, and 55.5. Anal.Calcd for C_23_H_17_BrN_2_O_5_: C, 57.40; H, 3.56; N, 5.82. Found: C, 57.47; H, 3.63; N, 5.88.

2-Amino-4-(3-((para-tolyl)oxy)phenyl)-6-(hydroxymethyl)-8-oxo-4,8-dihydropyrano[3,2-b]pyran-3-carbonitrile (6j).

Brown solid; Mp: 245–247 °C; IR (KBr): υ (cm^−1^) = 3431, 2200, 1634.

^1^H NMR (400 MHz, DMSO-*d*_6_) δ (ppm): 2.31 (s, 3H, -CH_3_), 4.10 (m, 2H, -CH_2_), 4.73 (s, 1H, -CH), 5.09 (s, 2H, -CH_2_), 5.69 (m, 1H, -CH_2_), 6.33 (s, 1H, -OH), 7.01 (d, 2H, Aromatic), 7.19 (m, 6H, Aromatic), 7.35 (d, 2H, -NH_2_). ^13^C NMR (101 MHz, DMSO-*d*_*6*_) δ (ppm): 169.6, 168.2, 159.1, 157.9, 149.2, 137.1, 136.1, 133.9, 132.9, 128.8, 127.8, 119.3, 115.0, 111.3, 69.1, 59.1, 55.9 and 20.8. Anal.Calcd for C_23_H_18_N_2_O_5_: C, 68.65; H, 4.51; N, 6.96. Found: C, 68.76; H, 4.67; N, 6.98.

2-Amino-4-(4-(benzyloxy)-3-methoxyphenyl)-6-(hydroxymethyl)-8-oxo-4,8 dihydropyrano[3,2-b]pyran-3-carbonitrile (6k).

Yellow solid; Mp: 234–237 °C; IR (KBr): υ (cm^−1^) = 3336, 2196, 1652, 1511. ^1^H NMR (400 MHz, DMSO-*d*_6_) δ (ppm): 4.13 (s, 3H, -CH_3_), 4.10 (m, 2H, -CH_2_), 4.75 (s, 1H, -CH), 5.07 (s, 2H, -CH_2_), 5.69 (m, 1H, -CH_2_), 6.34 (s, 1H, -OH), 6.77 (d, 1H, Aromatic), 6.77 ( s, 1H, Aromatic), 6.90 (d, 1H, Aromatic), 7.40 (d, 2H, -NH_2_), 7.43 (m, 5H, Aromatic). ^13^C NMR (101 MHz, DMSO-*d*_*6*_) δ (ppm): 169.6, 168.1, 159.2, 149.1, 147.5, 137.0, 136.1, 133.5, 128.4, 127.9, 119.7, 113.5, 111.6, 69.9, 59.1, and 55.6. Anal.Calcd for C_24_H_20_N_2_O_5_: C, 66.66; H, 4.66; N, 6.48. Found: C, 66.73; H, 4.57; N, 6.41.

2-Amino-4-(4-((4-fluorobenzyl)oxy)-3-methoxyphenyl)-6-(hydroxymethyl)-8-oxo-4,8-dihydropyrano[3,2-b]pyran-3-carbonitrile (6l).

Brown solid; Mp: 232–235 °C; IR (KBr): υ (cm^−1^) = 3320, 2200, 1658, 1590.^1^H NMR (400 MHz, DMSO-*d*_6_) δ (ppm): 3.77 (s, 3H, -CH_3_), 4.19 (s, 2H, -CH_3_), 4.25 (s, 1H, -CH), 4.75 (s, 2H, -CH_2_), 5.69 (m, 1H, -CH_2_), 6.34 (s, 1H, -OH), 6.77 (d, 1H, Aromatic), 6.79 ( s, 1H, Aromatic), 6.94 (d, 1H, Aromatic), 7.43 (m, 4H, Aromatic), 7.52 (d, 2H, -NH_2_). ^13^C NMR (101 MHz, DMSO-*d*_*6*_) δ (ppm): 169.8, 168.1, 162.9, 160.5, 159.2, 149.1, 147.3, 136.1, 133.6, 130.1, 119.7, 115.3, 113.5, 111.6, 69.1, 59.1, 55.6, and 55.5. Anal.Calcd for C_24_H_19_FN_2_O_5_: C, 64.00; H, 4.25; N, 6.22. Found: C, 63.98; H, 4.27; N, 6.18.

2-Amino-4-(4-((4-cholorobenzyl)oxy)-3-methoxyphenyl)-6-(hydroxymethyl)-8-oxo-4,8-dihydropyrano[3,2-b]pyran-3-carbonitrile (6m).

White solid; Mp: 210–213 °C; IR (KBr): υ (cm^−1^) = 3331, 2221, 1631, 1518. ^1^H NMR (400 MHz, DMSO-*d*_6_) δ (ppm): 3.77 (s, 3H, -CH_3_), 4.18 (s, 2H, -CH_3_), 4.75 (s, 1H, -CH), 5.07 (s, 2H, -CH_2_), 5.68 (m, 1H, -CH_2_), 6.33 (s, 1H, -OH), 6.76 (d, 1H, Aromatic), 6.79 ( s, 1H, Aromatic), 6.91 (d, 1H, Aromatic), 7.06 (s, 2H, -NH_2_), 7.48 (s, 4H, Aromatic). ^13^C NMR (101 MHz, DMSO-*d*_*6*_) δ (ppm): 169.6, 168.1, 159.2, 149.1, 147.2, 136.1, 133.6, 132.4, 129.6, 128.4, 119.7, 113.6, 116.7, 111.3, 69.0, 59.1, 55.6 and 55.5. Anal.Calcd for C_24_H_19_ClN_2_O_5_: C, 61.74; H, 4.10; N, 6.00. Found: C, 61.65; H, 4.17; N, 6.10.

2-Amino-4-(4-((4-boromobenzyl)oxy)-3-methoxyphenyl)-6-(hydroxymethyl)-8-oxo-4,8-dihydropyrano[3,2-b]pyran-3-carbonitrile (6n).

Yellow solid; Mp: 202–206 °C; IR (KBr): υ (cm^−1^) = 3331, 2221, 1631, 1518.^1^H NMR (400 MHz, DMSO-*d*_6_) δ (ppm): 3.77 (s, 3H, -CH_3_), 4.13 (s, 2H, -CH_3_), 4.23 (s, 1H, -CH), 4.75 (s, 2H, -CH_2_), 5.69 (m, 1H, -CH_2_), 6.33 (s, 1H, -OH), 6.76 (d, 1H, Aromatic), 6.79 ( s, 1H, Aromatic), 6.90 (d, 1H, Aromatic), 7.05 (s, 2H, -NH_2_), 7.42 (d, 2H, Aromatic), 7.60 (d, 2H, Aromatic). ^13^C NMR (101 MHz, DMSO-*d*_*6*_) δ (ppm): 169.6, 168.1, 159.2, 149.1, 147.2, 136.5, 133.7, 131.3, 129.9, 120.9, 120.9, 119.7, 113.6, 111.6, 69.1, 59.1, and 55.6. Anal.Calcd for C_24_H_19_BrN_2_O_5_: C, 61.74; H, 4.10; N, 6.00. Found: C, 61.65; H, 4.17; N, 6.10.

2-Amino-6-(hydroxymethyl)-4-(3-methoxy-4-((4-methylbenzyl)oxy)phenyl)-8-oxo-4,8 dihydropyrano[3,2-b]pyran-3-carbonitrile (6o).

Brown solid; Mp: 241–245 °C; IR (KBr): υ (cm^−1^) = 3345, 2200, 1643, 1500. ^1^H NMR (400 MHz, DMSO-*d*_6_) δ (ppm): 2.33 (s, 3H, -CH_3_), 3.76 (s, 3H, -CH_3_), 4.18 (s, 2H, -CH_3_), 4.75 (s, 1H, -CH), 5.01 (s, 2H, -CH_2_), 5.73 (m, 1H, -CH_2_), 6.33 (s, 1H, -OH), 6.76 (d, 1H, Aromatic), 6.79 ( s, 1H, Aromatic), 6.91 (d, 1H, Aromatic), 7.06 (s, 2H, -NH_2_), 7.48 (s, 4H, Aromatic). ^13^C NMR (101 MHz, DMSO-*d*_*6*_) δ (ppm): 169.6, 168.1, 159.2, 149.1, 147.4, 137.1, 136.1, 133.9, 128.9, 127.9, 119.6, 113.4, 111.6, 111.3, 69.7, 59.1, 55.6, 20.8. Anal.Calcd for C_25_H_22_N_2_O_5_: C, 67.26; H, 4.97; N, 6.27. Found: C, 67.31; H, 5.07; N, 6.33.

### Tyrosinase inhibition assay

We conducted the assay of mushroom tyrosinase (EC 1.14.18.1) utilizing L-Dopa as the substrate, as previously described, with minor adaptations [24]. We employed a spectrophotometric technique to assess the enzyme's diphenolase activity, observing dopachrome formation at 490 nm. All compounds being tested and kojic acid were initially dissolved in DMSO and diluted to the desired levels. In a 96-well microplate, 10 µl of the test samples were combined with 140 µl of 50 mM phosphate buffer (pH = 6.8). Subsequently, 20 µl of tyrosinase (273 U mL^−1^) was introduced. Following a pre-incubation period of 20 min at 28 °C, 20 µl of L-Dopa solution (resulting in a final concentration of 0.7 mM) was added to the mixture. Following a 10-min incubation, the absorbance of the samples was measured. The inhibitory activity of the tested compounds was stated as IC_50_. The ratio percentage of inhibition was calculated according to the equation below:

$${\text{Inhibition }}\left( \% \right) \, = { 1}00 \, \left( {{\text{Abs}}_{{{\text{control}}}} - {\text{ Abs}}_{{{\text{compound}}}} } \right)/{\text{Abs}}_{{{\text{control}}}}$$

### Determination of the inhibition type

For the purpose of kinetic analysis, we focused on **6b**, the most potent derivative. The inhibitor concentrations employed ranged from 2 to 20 μM, while the substrate (L-Dopa) concentrations in the rane of 0.1 mM to 1.5 mM through all kinetic experiments. The pre-incubation and measurement durations followed the protocol outlined in the mushroom tyrosinase inhibition assay [24]. The maximum initial velocity was ascertained from the initial linear segment of absorbance within a 10-min window following the introduction of L-Dopa, with measurements taken at 1-min intervals. Utilizing the Lineweaver–Burk plot technique, we calculated the Michaelis constant (*K*_*m*_) and the tyrosinase activity's maximal velocity (*V*_*max*_). This plot involved various L-Dopa concentrations as the substrate. For assessing the enzyme's inhibition type, we employed Lineweaver–Burk plots featuring the reciprocal of velocities (1/V) plotted against the reciprocal of substrate concentrations (1/[S] mM^−1^) [24].

### Molecular docking study

According to previously reported procedures, an induced fit docking study (IFD) was performed using Maestro Molecular Modeling platform to understand the interaction mode of the most potent compound over tyrosinase enzyme with PDB ID of 2Y9X with the grid center of X = − 8.06, Y = − 25.78, and Z = − 39.39 [25, 26].

### Molecular dynamics simulations

Molecular dynamics simulation was performed using the Desmond v5.3 module implemented in the Maestro interface (from Schrödinger 2018‐4 suite). The appropriate pose for the molecular dynamics simulation procedure of compound **6b** was achieved by the IFD method.

An molecular dynamics simulation of compound **6b** was performed using the Desmond v5.3 module in the Maestro interface. The system was prepared by solvating the protein–ligand complex with explicit water molecules and adding counter-ions and NaCl to achieve cellular ionic concentrations. The molecular dynamics protocol involved minimization, pre-production, and production molecular dynamics steps. The simulations were carried out in the NPT ensemble with constant pressure and temperature. The system underwent 30 ns of molecular dynamics simulation, and the dynamic behavior and structural changes were analyzed using the RMSD. The protein–ligand interactions were investigated using the energy-minimized structure from the equilibrated trajectory [25, 26].

### In silico prediction of drug-likeness and ADMET properties

The number of hydrogen bond acceptor (HBA) and donor (HBD), logP values, the topological polar surface area (tPSA), and the rotatable bond count (RBCs) of the selected compounds were calculated using the SwissADME and pkCSM online softwares. ADMET prediction of the selected compounds was performed by online software pkCSM [22, 23].

### Supplementary Information

**Additional file 1**: **Fig. S1**. FT-IR, ^1^H NMR and ^13^C NMR spectrums of 2-amino-4-(4-(benzyloxy)phenyl)-6-(hydroxymethyl)-8-oxo-4,8-dihydropyrano [3,2-b] pyran-3-carbonitrile (6a). **Fig. S2**. FT-IR, ^1^H NMR and ^13^C NMR spectrums of 2-amino-4-(4-((4-fluorobenzyl)oxy)phenyl)-6-(hydroxymethyl)-8-oxo-4,8-dihydropyrano[3,2-b]pyran-3-carbonitrile (6b). **Fig. S3**. FT-IR, ^1^H NMR and ^13^C NMR spectrums of 2-amino-4-(4-((4-chlorobenzyl)oxy)phenyl)-6-(hydroxymethyl)-8-oxo-4,8-dihydropyrano[3,2-b]pyran-3-carbonitrile (6c). **Fig. S4**. FT-IR, ^1^H NMR and ^13^C NMR spectrums of 2-amino-4-(4-((4-boromobenzyl)oxy)phenyl)-6-(hydroxymethyl)-8-oxo-4,8 dihydropyrano[3,2-b]pyran-3-carbonitrile (6d). **Fig. S5**. FT-IR, ^1^H NMR and ^13^C NMR spectrums of 2-amino-6-(hydroxymethyl)-4-(4-((4-methylbenzyl)oxy)phenyl)-8-oxo-4,8-dihydropyrano[3,2-b]pyran-3-carbonitrile (6e). **Fig. S6**. FT-IR, ^1^H NMR and ^13^C NMR spectrums of 2-amino-4-(3-(benzyloxy)phenyl)-6-(hydroxymethyl)-8-oxo-4,8-dihydropyrano[3,2-b]pyran-3-carbonitrile (6f). **Fig. S7**. FT-IR, ^1^H NMR and ^13^C NMR spectrums of 2-amino-4-(3-((4-fluorobenzyl)oxy)phenyl)-6-(hydroxymethyl)-8-oxo-4,8-dihydropyrano[3,2-b]pyran-3-carbonitrile (6g). **Fig. S8**. FT-IR, ^1^H NMR and ^13^C NMR spectrums of 2-amino-4-(3-((4-chlorobenzyl)oxy)phenyl)-6-(hydroxymethyl)-8-oxo-4,8-dihydropyrano[3,2-b]pyran-3-carbonitrile (6h). **Fig. S9**. FT-IR, ^1^H NMR and ^13^C NMR spectrums of 2-amino-4-(3-((4-boromobenzyl)oxy)phenyl)-6-(hydroxymethyl)-8-oxo-4,8-dihydropyrano[3,2-b]pyran-3-carbonitrile (6i), **Fig. S10**. FT-IR, ^1^H NMR and ^13^C NMR spectrums of 2-amino-4-(3-((4-boromobenzyl)oxy)phenyl)-6-(hydroxymethyl)-8-oxo-4,8-dihydropyrano[3,2-b]pyran-3-carbonitrile (6j). **Fig. S11**. FT-IR, ^1^H NMR and ^13^C NMR spectrums of 2-amino-4-(4-(benzyloxy)-3-methoxyphenyl)-6-(hydroxymethyl)-8-oxo-4,8-dihydropyrano[3,2-b]pyran-3-carbonitrile (6k), **Fig. S12**. FT-IR, ^1^H NMR and ^13^C NMR spectrums of 2-amino-4-(4-((4-fluorobenzyl)oxy)-3-methoxyphenyl)-6-(hydroxymethyl)-8-oxo-4,8-dihydropyrano[3,2-b]pyran-3-carbonitrile (6l), **Fig. S13**. FT-IR, ^1^H NMR and ^13^C NMR spectrums of 2-amino-4-(4-((4-cholorobenzyl)oxy)-3-methoxyphenyl)-6-(hydroxymethyl)-8-oxo-4,8-dihydropyrano[3,2-b]pyran-3-carbonitrile (6m). **Fig. S14**. FT-IR, ^1^H NMR and ^13^C NMR spectrums of 2-amino-4-(4-((4-boromobenzyl)oxy)-3-methoxyphenyl)-6-(hydroxymethyl)-8-oxo-4,8-dihydropyrano[3,2-b]pyran-3-carbonitrile (6n). **Fig. S15**. FT-IR, ^1^H NMR and ^13^C NMR spectrums of 2-amino-6-(hydroxymethyl)-4-(3-methoxy-4-((4-methylbenzyl)oxy)phenyl)-8-oxo-4,8 dihydropyrano[3,2-b]pyran-3-carbonitrile (6o).

## Data Availability

This published article and its additional file includes some data generated or analyzed during this study. Other datasets used and analyzed during the current study are available from the corresponding author on reasonable request.

## References

[1] He X, Jin S, Dai X, Chen L, Xiang L, Zhang C (2023). The emerging role of visible light in melanocyte biology and skin pigmentary disorders: friend or foe?. J Clin Med.

[2] Slominski RM, Sarna T, Płonka PM, Raman C, Brożyna AA, Slominski AT (2022). Melanoma, melanin, and melanogenesis: the yin and yang relationship. Front Oncol.

[3] Solano F (2018). On the metal cofactor in the tyrosinase family. Int J Mol Sci.

[4] Li J, Feng L, Liu L, Wang F, Ouyang L, Zhang L (2021). Recent advances in the design and discovery of synthetic tyrosinase inhibitors. Eur J Med Chem.

[5] He M, Fan M, Yang W, Peng Z, Wang G (2023). Novel kojic acid-1,2,4-triazine hybrids as anti-tyrosinase agents: synthesis, biological evaluation, mode of action, and anti-browning studies. Food Chem.

[6] Marieshwari BN, Bhuvaragavan S, Sruthi K, Mullainadhan P, Janarthanan S (2023). Insect phenoloxidase and its diverse roles: melanogenesis and beyond. J Comp Physiol B.

[7] Nagatsu T, Nakashima A, Watanabe H, Ito S, Wakamatsu K (2022). Neuromelanin in Parkinson's disease: tyrosine hydroxylase and tyrosinase. Int J Mol Sci.

[8] Carballo-Carbajal I, Laguna A, Romero-Giménez J, Cuadros T, Bové J, Martinez-Vicente M (2019). Brain tyrosinase overexpression implicates age-dependent neuromelanin production in Parkinson’s disease pathogenesis. Nat Commun.

[9] Chen YM, Li C, Zhang WJ, Shi Y, Wen ZJ, Chen QX (2019). Kinetic and computational molecular docking simulation study of novel kojic acid derivatives as anti-tyrosinase and antioxidant agents. J Enzyme Inhib Med Chem.

[10] Yamada R, Yoshie T, Wakai S, Asai-Nakashima N, Okazaki F, Ogino C (2014). Aspergillus oryzae-based cell factory for direct kojic acid production from cellulose. Microb Cell Fact.

[11] Wang W, Gao Y, Wang W, Zhang J, Yin J, Le T (2022). Kojic acid showed consistent inhibitory activity on tyrosinase from mushroom and in cultured B16F10 cells compared with arbutins. Antioxidants.

[12] Xie W, Zhang J, Ma X, Yang W, Zhou Y, Tang X (2015). Synthesis and biological evaluation of kojic acid derivatives containing 1, 2, 4-triazole as potent tyrosinase inhibitors. Chem Biol Drug Des.

[13] Ashooriha M, Khoshneviszadeh M, Khoshneviszadeh M, Moradi SE, Rafiei A, Kardan M (2019). 1,2,3-Triazole-based kojic acid analogs as potent tyrosinase inhibitors: design, synthesis and biological evaluation. Bioorgan Chem.

[14] Lee YS, Park JH, Kim MH, Seo SH, Kim HJ (2006). Synthesis of tyrosinase inhibitory kojic acid derivative. Arch Pharm.

[15] Xie W, Zhang H, He J, Zhang J, Yu Q, Luo C (2017). Synthesis and biological evaluation of novel hydroxybenzaldehyde-based kojic acid analogues as inhibitors of mushroom tyrosinase. Bioorgan Med Chem Lett.

[16] Ranjbar S, Razmara S, Khademian S, Malekzadeh Z, Kabiri M, Ghasemi Y (2023). 6-(Hydroxymethyl)-8-oxo-4, 8-dihydropyrano [3, 2-b] pyrans as new tyrosinase inhibitors and antioxidant agents. ChemistrySelect.

[17] Debbabi M, Nimbarte VD, Chekir S, Chortani S, Romdhane A, Ben jannet H. (2019). Design and synthesis of novel potent anticoagulant and anti-tyrosinase pyranopyrimidines and pyranotriazolopyrimidines: Insights from molecular docking and SAR analysis. Bioorgan Chem.

[18] Najafi Z, Alaei M, Bahmani A, Akbarzadeh T, Hariri R, Chehardoli G (2023). Fused 1,4-Dihydropyridines and their corresponding pyridines: synthesis, molecular modeling and cholinesterase inhibition. ChemistrySelect.

[19] Najafi Z, Ebadi A, Chehardoli G, Ziaei M, khoshneviszadeh M, Akbarzadeh T, (2023). Design, synthesis, in vitro, and in silico studies of novel benzylidene 6-methoxy-1-tetralone linked to benzyloxy and benzyl -1,2,3- triazole rings as potential tyrosinase inhibitors. J Mol Struct.

[20] Kurosawa W, Kan T, Fukuyama T (2003). Stereocontrolled total synthesis of (−)-Ephedradine A (Orantine). J Am Chem Soc.

[21] Elinson MN, Ryzhkov FV, Nasybullin RF, Vereshchagin AN, Egorov MP (2016). Fast efficient and general PASE approach to medicinally relevant 4H,5H-Pyrano-[4,3-b]pyran-5-one and 4,6-Dihydro-5H-pyrano-[3,2-c]pyridine-5-one scaffolds. Helv Chim Acta.

[22] Pires DEV, Blundell TL, Ascher DB (2015). pkCSM: predicting small-molecule pharmacokinetic and toxicity properties using graph-based signatures. J Med Chem.

[23] Daina A, Michielin O, Zoete V (2017). SwissADME: a free web tool to evaluate pharmacokinetics, drug-likeness and medicinal chemistry friendliness of small molecules. Sci Rep.

[24] Hosseinpoor H, Moghadam Farid S, Iraji A, Askari S, Edraki N, Hosseini S (2021). Anti-melanogenesis and anti-tyrosinase properties of aryl-substituted acetamides of phenoxy methyl triazole conjugated with thiosemicarbazide: Design, synthesis and biological evaluations. Bioorg Chem.

[25] Ghasemi N, Moradi S, Iraji A, Mahdavi M (2023). Thiazolopyrimidine derivatives as novel class of small molecule tyrosinase inhibitor. BMC Chemistry.

[26] Iraji A, Sheikhi N, Attarroshan M, Reaz Sharifi Ardani G, Kabiri M, Naghibi Bafghi A, Kobarfard F, Rezaei Z, Khoshneviszadeh M, Foroumadi A, Mirfazli SS (2022). Design, synthesis, spectroscopic characterization, in vitro tyrosinase inhibition, antioxidant evaluation, in silico and kinetic studies of substituted indole-carbohydrazides. Bioorgan Chem.

