# Supplementary material for: Design, synthesis, and molecular dynamics simulation studies of some novel kojic acid fused 2-amino-3-cyano-4H-pyran derivatives as tyrosinase inhibitors
Source: BMC Chem. 2024 Feb 22;18(1):41. doi: 10.1186/s13065-024-01134-1 (PMC10885651; doi:10.1186/s13065-024-01134-1)
Supplement: Supplementary file 1 — Additional file 1: Fig. S1. FT-IR, 1H NMR and 13C NMR spectrums of 2-amino-4-(4-(benzyloxy)phenyl)-6-(hydroxymethyl)-8-oxo-4,8-dihydropyrano [3,2-b] pyran-3-carbonitrile (6a). Fig. S2. FT-IR, 1H NMR and 13C NMR spectrums of 2-amino-4-(4-((4-fluorobenzyl)oxy)phenyl)-6-(hydroxymethyl)-8-oxo-4,8-dihydropyrano[3,2-b]pyran-3-carbonitrile (6b). Fig. S3. FT-IR, 1H NMR and 13C NMR spectrums of 2-amino-4-(4-((4-chlorobenzyl)oxy)phenyl)-6-(hydroxymethyl)-8-oxo-4,8-dihydropyrano[3,2-b]pyran-3-carbonitrile (6c). Fig. S4. FT-IR, 1H NMR and 13C NMR spectrums of 2-amino-4-(4-((4-boromobenzyl)oxy)phenyl)-6-(hydroxymethyl)-8-oxo-4,8 dihydropyrano[3,2-b]pyran-3-carbonitrile (6d). Fig. S5. FT-IR, 1H NMR and 13C NMR spectrums of 2-amino-6-(hydroxymethyl)-4-(4-((4-methylbenzyl)oxy)phenyl)-8-oxo-4,8-dihydropyrano[3,2-b]pyran-3-carbonitrile (6e). Fig. S6. FT-IR, 1H NMR and 13C NMR spectrums of 2-amino-4-(3-(benzyloxy)phenyl)-6-(hydroxymethyl)-8-oxo-4,8-dihydropyrano[3,2-b]pyran-3-carbonitrile (6f). Fig. S7. FT-IR, 1H NMR and 13C NMR spectrums of 2-amino-4-(3-((4-fluorobenzyl)oxy)phenyl)-6-(hydroxymethyl)-8-oxo-4,8-dihydropyrano[3,2-b]pyran-3-carbonitrile (6g). Fig. S8. FT-IR, 1H NMR and 13C NMR spectrums of 2-amino-4-(3-((4-chlorobenzyl)oxy)phenyl)-6-(hydroxymethyl)-8-oxo-4,8-dihydropyrano[3,2-b]pyran-3-carbonitrile (6h). Fig. S9. FT-IR, 1H NMR and 13C NMR spectrums of 2-amino-4-(3-((4-boromobenzyl)oxy)phenyl)-6-(hydroxymethyl)-8-oxo-4,8-dihydropyrano[3,2-b]pyran-3-carbonitrile (6i), Fig. S10. FT-IR, 1H NMR and 13C NMR spectrums of 2-amino-4-(3-((4-boromobenzyl)oxy)phenyl)-6-(hydroxymethyl)-8-oxo-4,8-dihydropyrano[3,2-b]pyran-3-carbonitrile (6j). Fig. S11. FT-IR, 1H NMR and 13C NMR spectrums of 2-amino-4-(4-(benzyloxy)-3-methoxyphenyl)-6-(hydroxymethyl)-8-oxo-4,8-dihydropyrano[3,2-b]pyran-3-carbonitrile (6k), Fig. S12. FT-IR, 1H NMR and 13C NMR spectrums of 2-amino-4-(4-((4-fluorobenzyl)oxy)-3-methoxyphenyl)-6-(hydroxymethyl)-8-oxo-4,8-dihydropyrano[3,2-b]pyran-3-carbonitrile (6l), Fig. S [file 13065_2024_1134_MOESM1_ESM.docx]

**Design, synthesis, and molecular dynamics simulation studies of** **some novel kojic acid fused 2-amino-3-cyano-4*H*-pyran derivatives as tyrosinase inhibitors**

Zahra Najafi,^a*^ Maryam Zandi Haramabadi,^a^ Gholamabbas Chehardoli,^b^ Ahmad Ebadi‬^b^

^a^ Department of Medicinal Chemistry, School of Pharmacy, Hamadan University of Medical Sciences, Hamadan, Iran.

^b^ Department of Medicinal Chemistry, School of Pharmacy, Medicinal Plants and Natural Products Research Center, Hamadan University of Medical Sciences, Hamadan, Iran.

^c^ Stem Cells Technology Research Center, Shiraz University of Medical Sciences, Shiraz, Iran.

^d^ Research Center for Traditional Medicine and History of Medicine, Department of Persian Medicine, School of Medicine, Shiraz University of Medical Sciences, Shiraz, Iran.

*Corresponding authors:

E-mail [najafi.zch@gmail.com](mailto:najafi.zch@gmail.com);z.najafi@umsha.ac.ir

and aida. iraji@gmail.com; iraji@sums.ac.ir

**Fig. S1.** **FT-IR, ^1^H NMR and ^13^C NMR spectrums of 2-amino-4-(4-(benzyloxy) phenyl)-6-(hydroxymethyl)-8-oxo-4, 8-dihydropyrano [3, 2-*b*] pyran-3-carbonitrile (6a).**

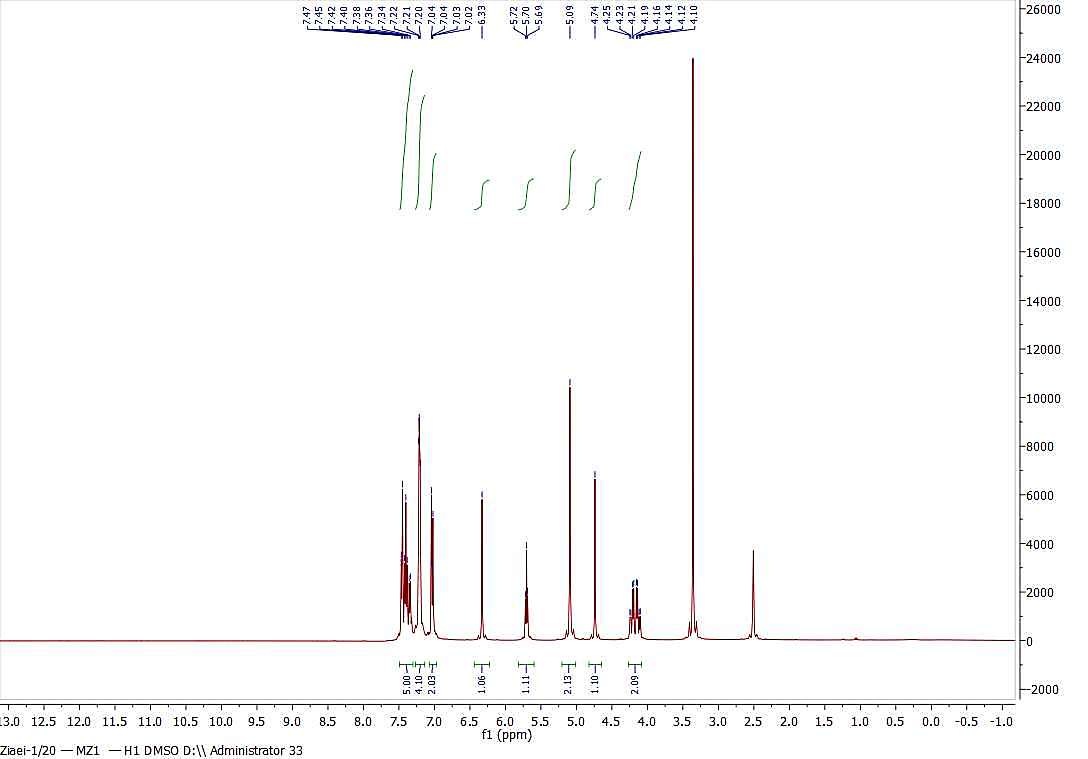


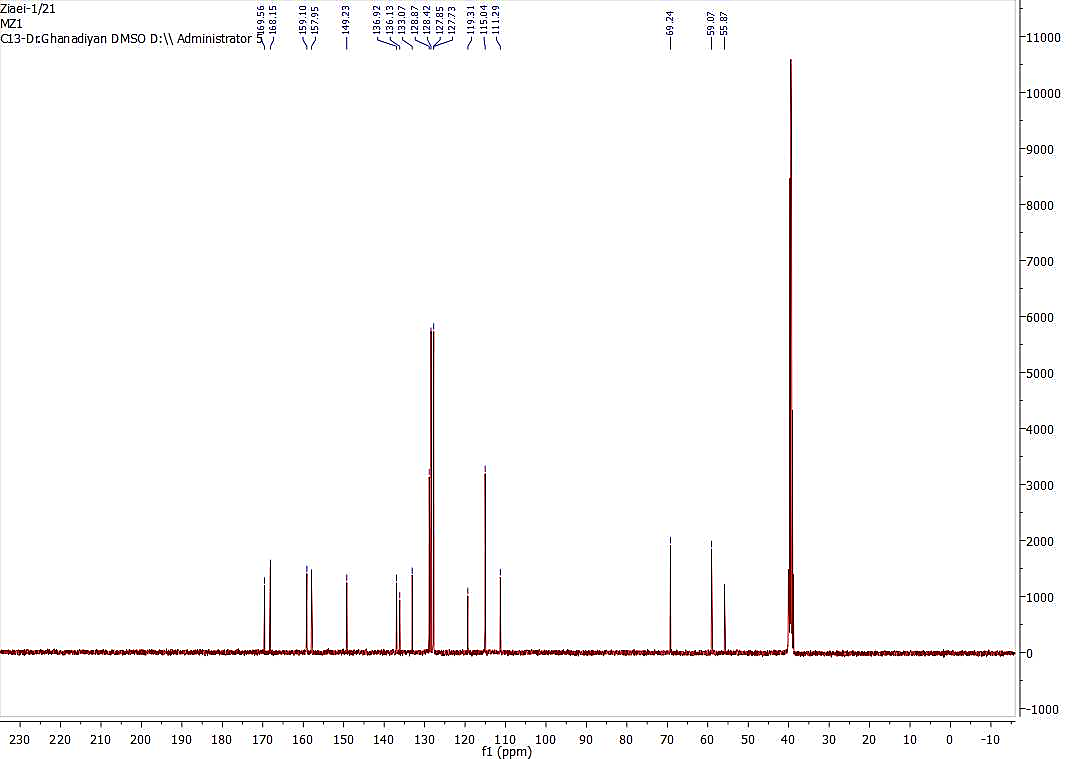


**Fig. S2.** **FT-IR, ^1^H NMR and ^13^C NMR spectrums of 2-amino-4-(4-((4-fluorobenzyl)oxy)phenyl)-6-(hydroxymethyl)-8-oxo-4,8-dihydropyrano[3,2-b]pyran-3-carbonitrile (6b).**

**
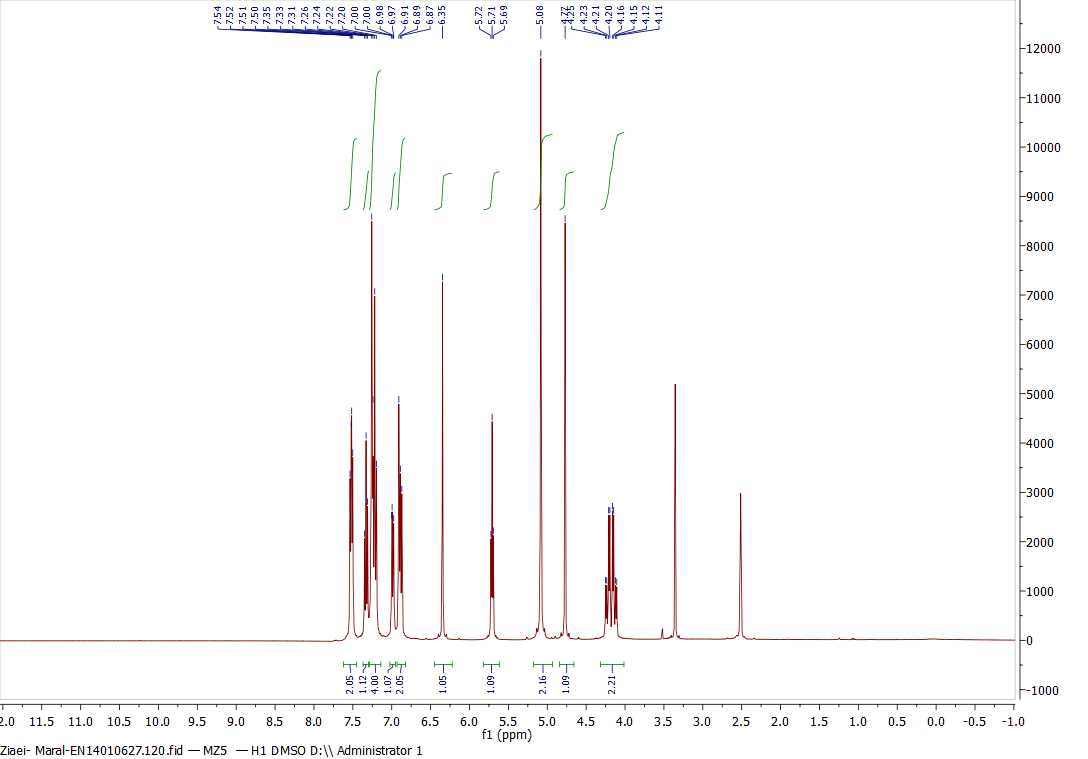
**


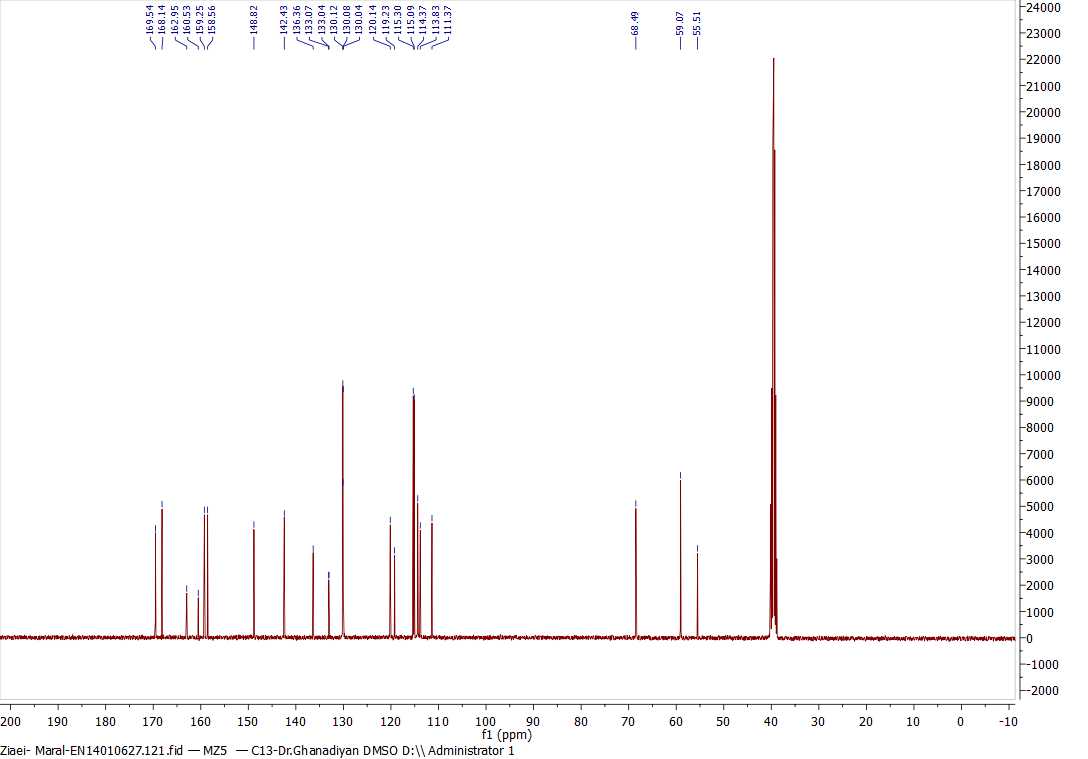


**Fig. S3.** **FT-IR, ^1^H NMR and ^13^C NMR spectrums of 2-amino-4-(4-((4-chlorobenzyl)oxy)phenyl)-6-(hydroxymethyl)-8-oxo-4,8 dihydropyrano[3,2-b]pyran-3-carbonitrile (6c).**

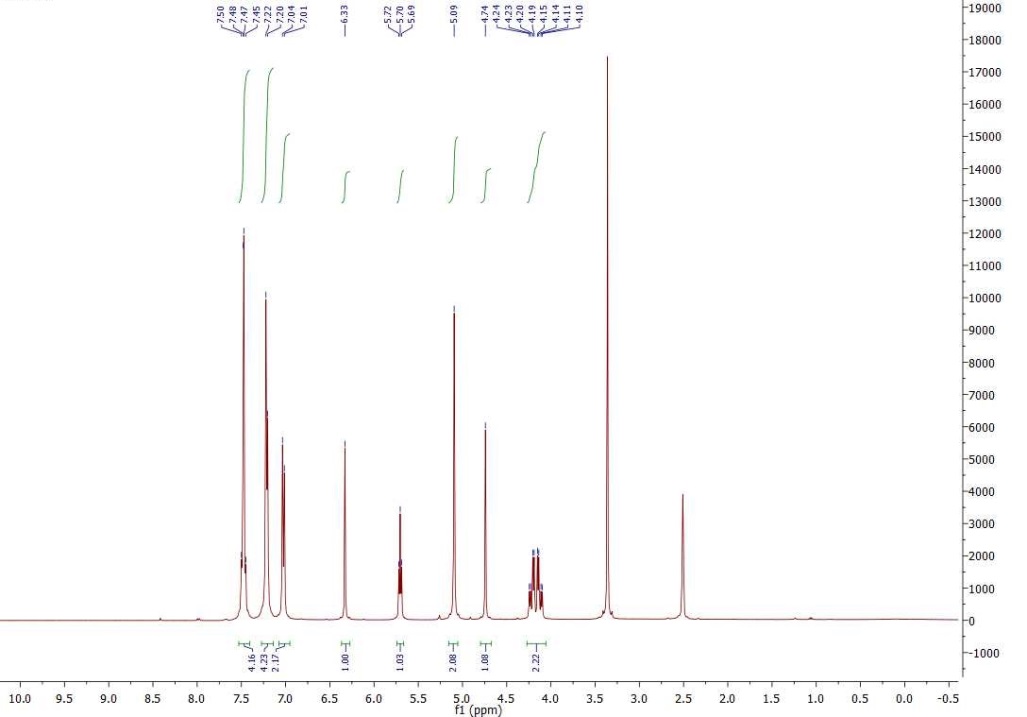


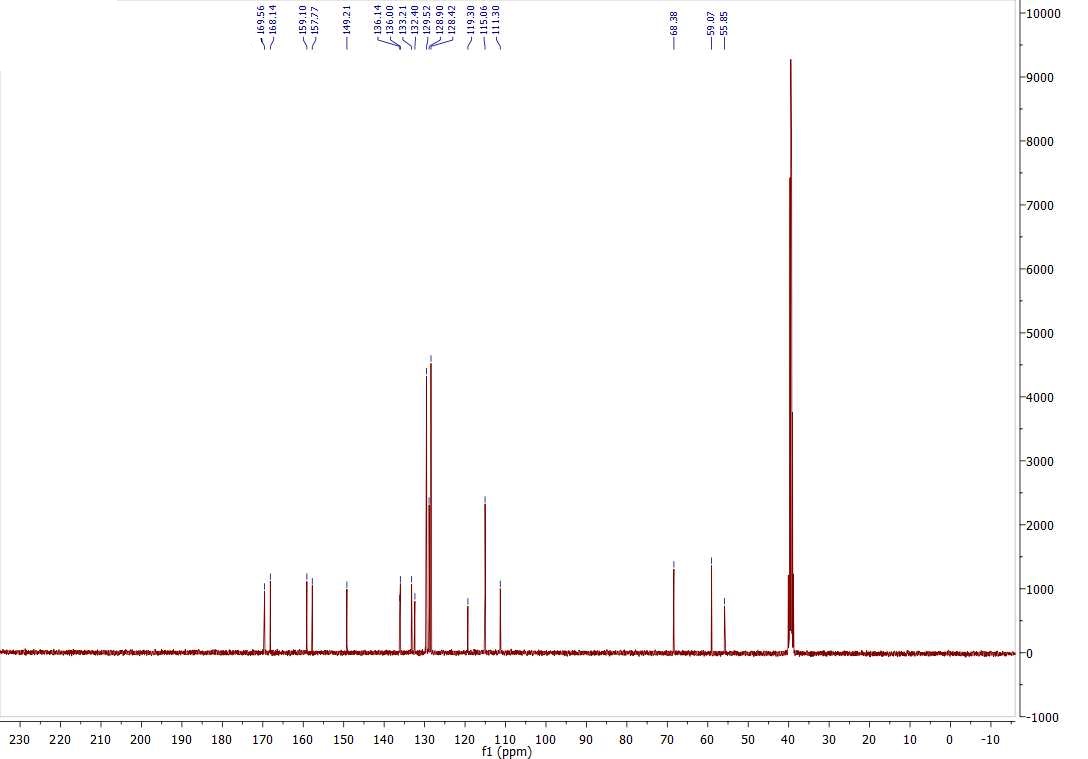


**Fig. S4. FT-IR, ^1^H NMR and ^13^C NMR spectrums of 2-amino-4-(4-((4-boromobenzyl)oxy)phenyl)-6-(hydroxymethyl)-8-oxo-4,8 dihydropyrano[3,2-b]pyran-3-carbonitrile (6d).**

**
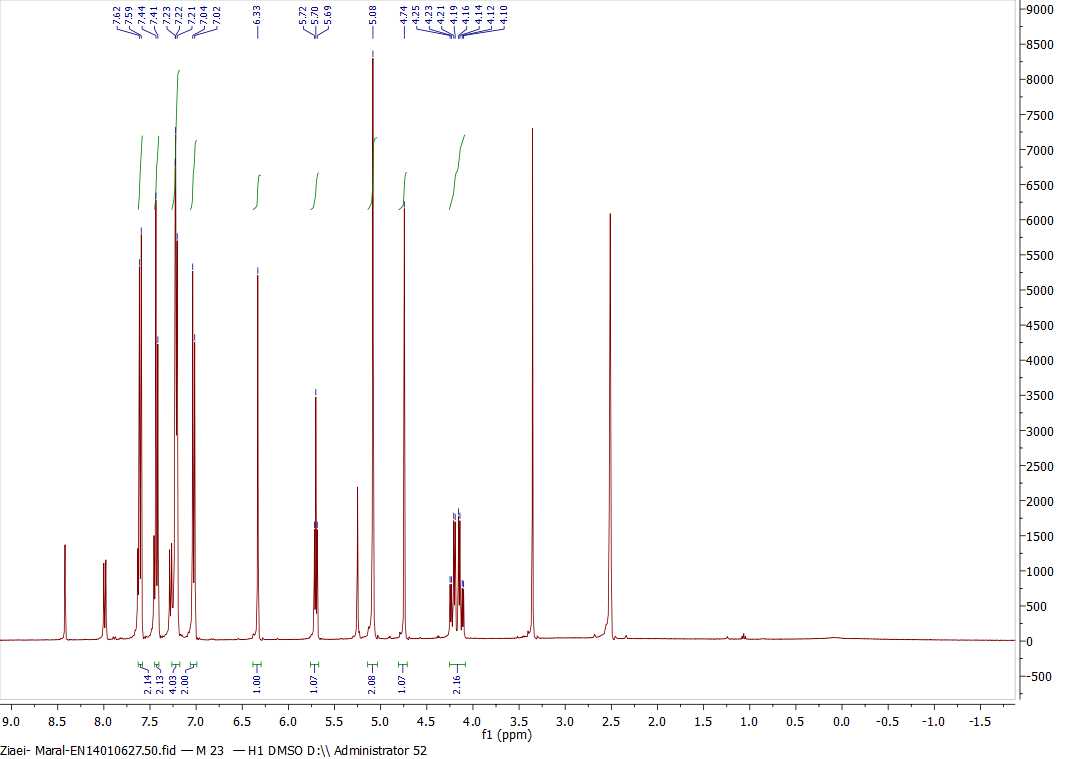
**


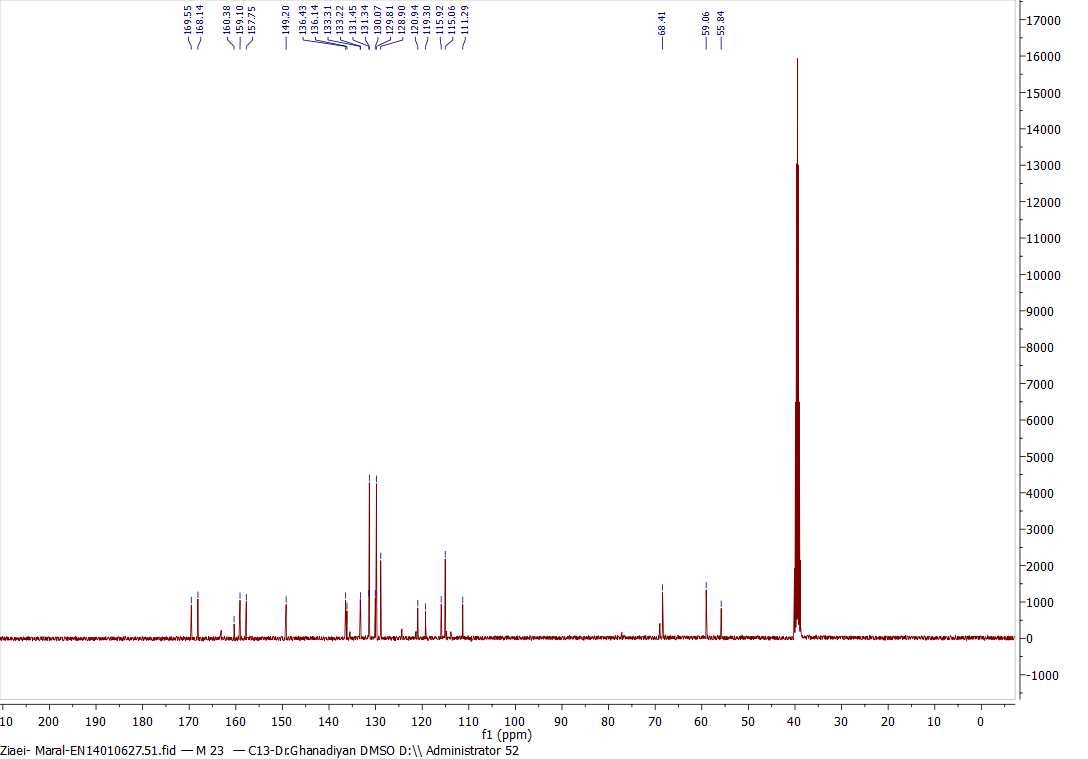


**Fig. S5. FT-IR, ^1^H NMR and ^13^C NMR spectrums of 2-amino-6-(hydroxymethyl)-4-(4-((4-methylbenzyl)oxy)phenyl)-8-oxo-4,8-dihydropyrano[3,2-b]pyran-3-carbonitrile (6e).**

**
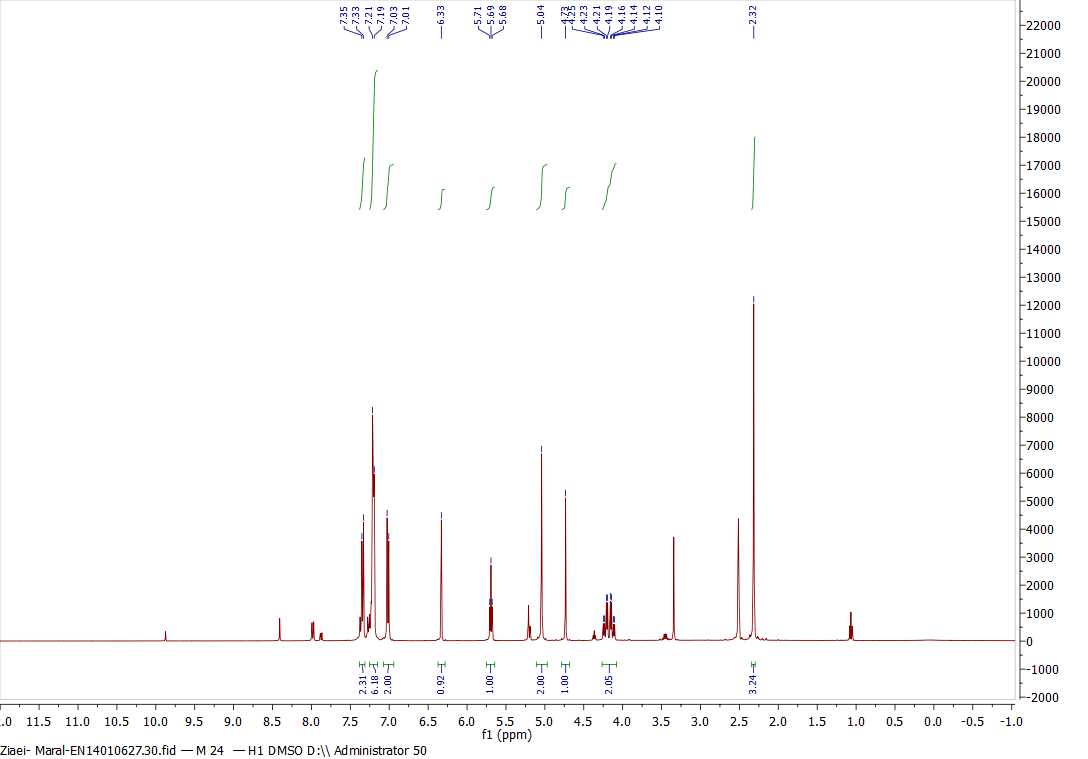
**

**
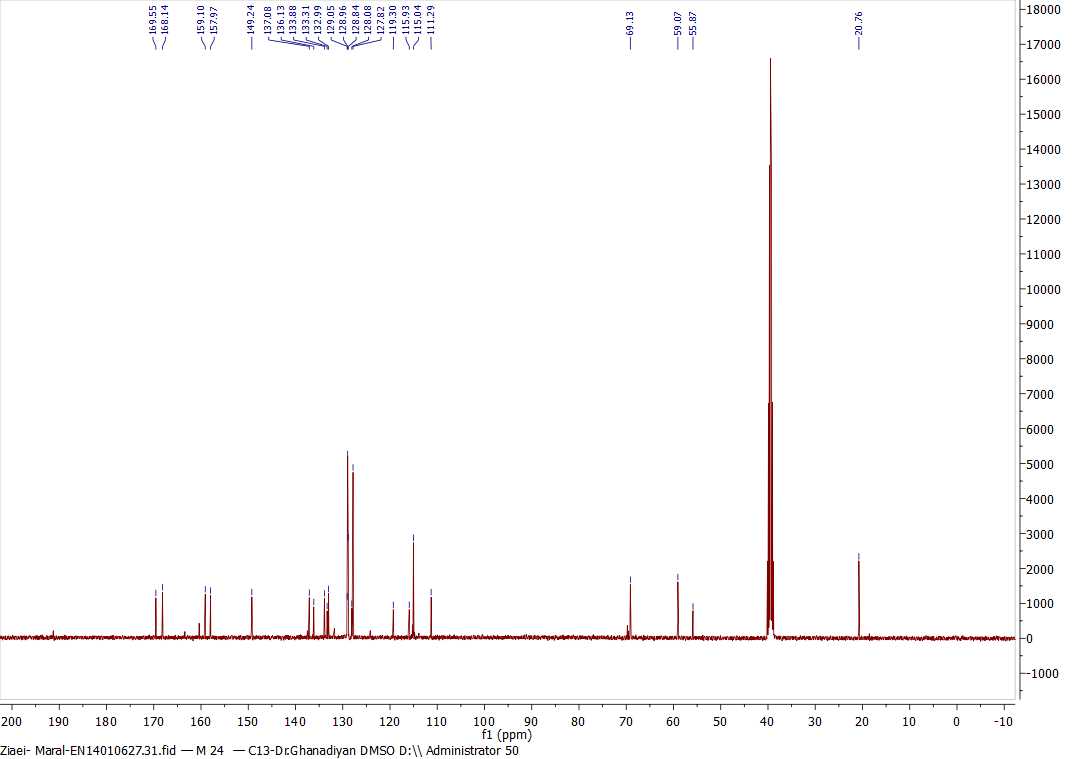
**

**Fig. S6. FT-IR, ^1^H NMR and ^13^C NMR spectrums of 2-amino-4-(3-(benzyloxy)phenyl)-6-(hydroxymethyl)-8-oxo-4,8-dihydropyrano[3,2-b]pyran-3-carbonitrile (6f).**

**
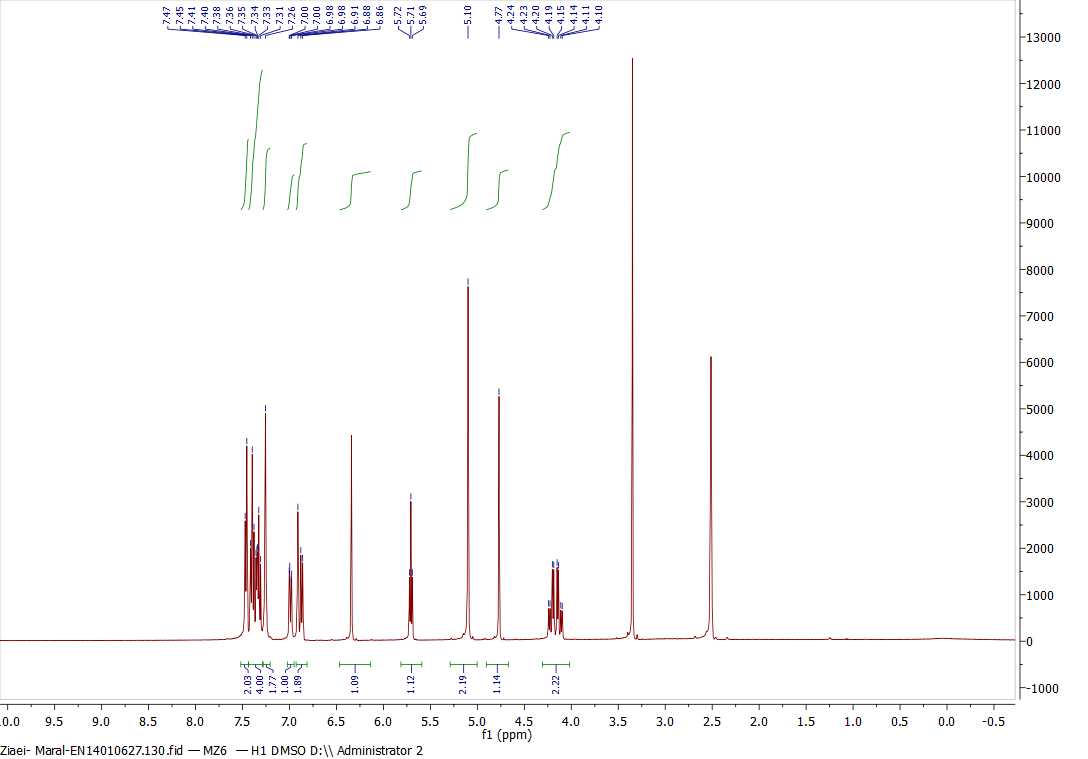
**

**
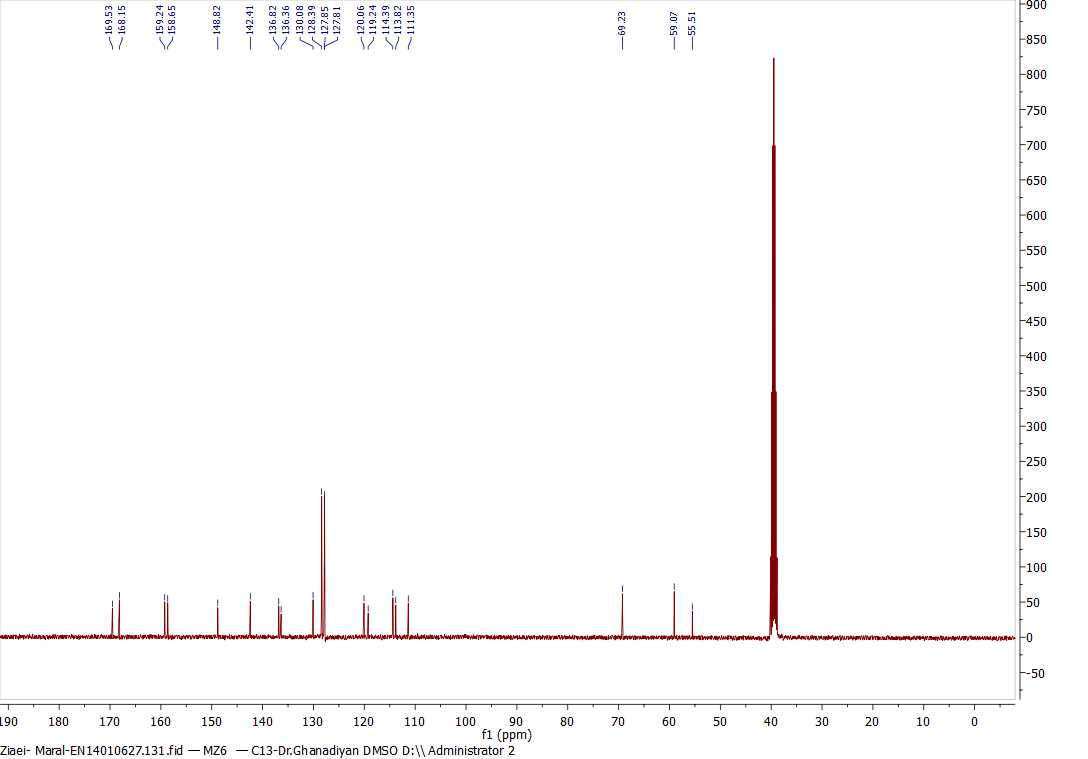
**

**Fig. S7. FT-IR, ^1^H NMR and ^13^C NMR spectrums of 2-amino-4-(3-((4-fluorobenzyl)oxy)phenyl)-6-(hydroxymethyl)-8-oxo-4,8-dihydropyrano[3,2-b]pyran-3-carbonitrile (6g).**

**
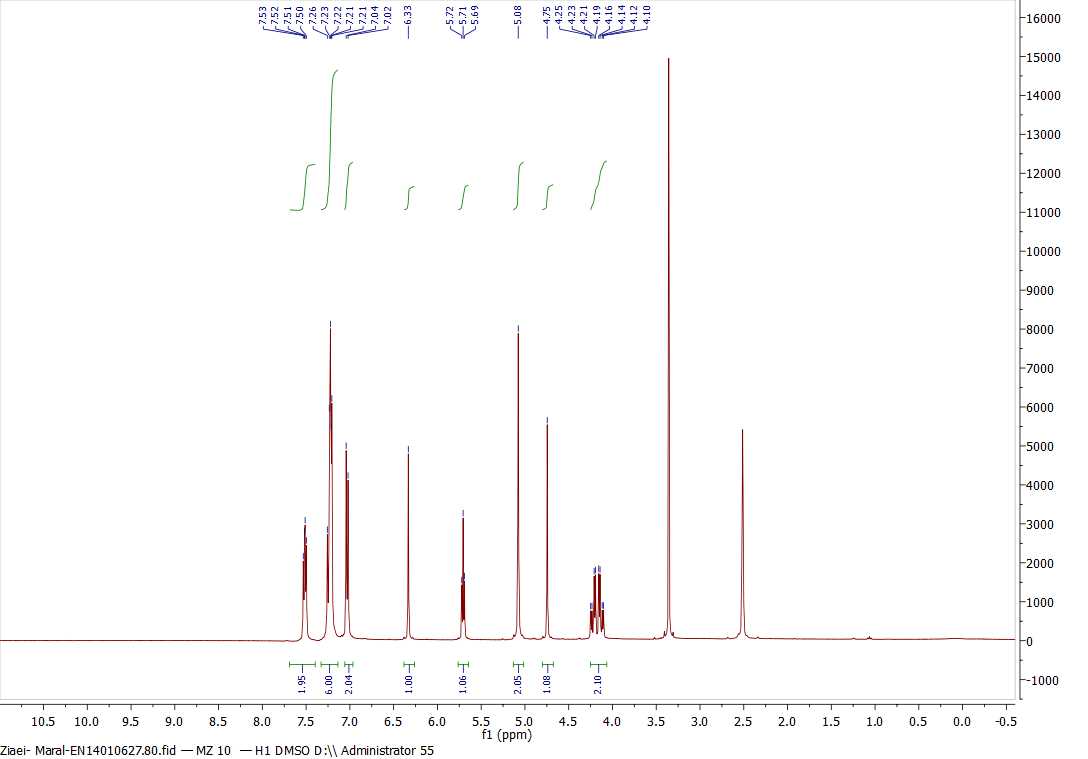
**

**
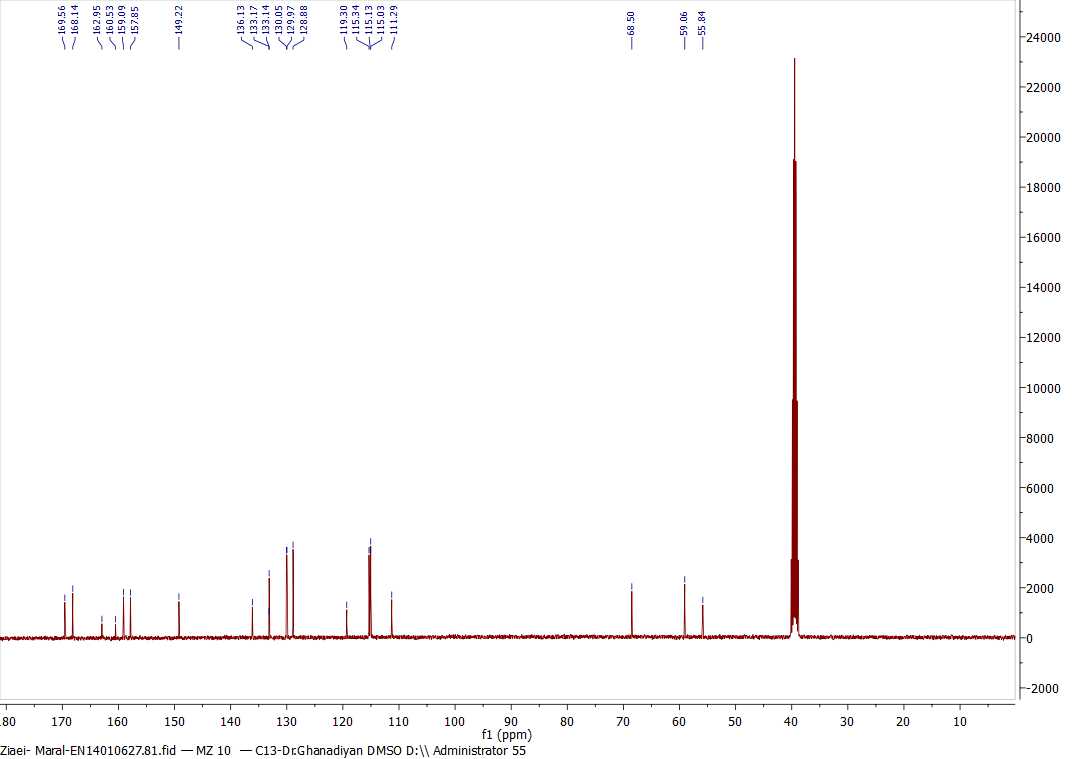
**

**Fig. S8. FT-IR, ^1^H NMR and ^13^C NMR spectrums of 2-amino-4-(3-((4-chlorobenzyl)oxy)phenyl)-6-(hydroxymethyl)-8-oxo-4,8-dihydropyrano[3,2-b]pyran-3-carbonitrile (6h).**

**
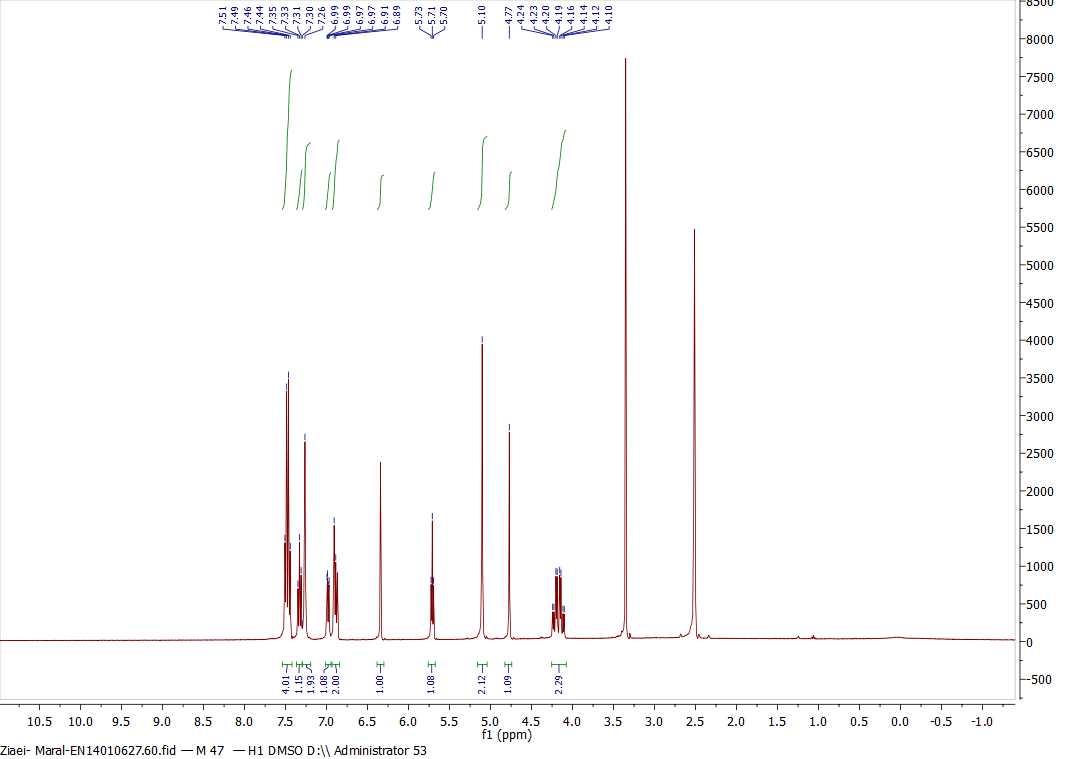
**

**
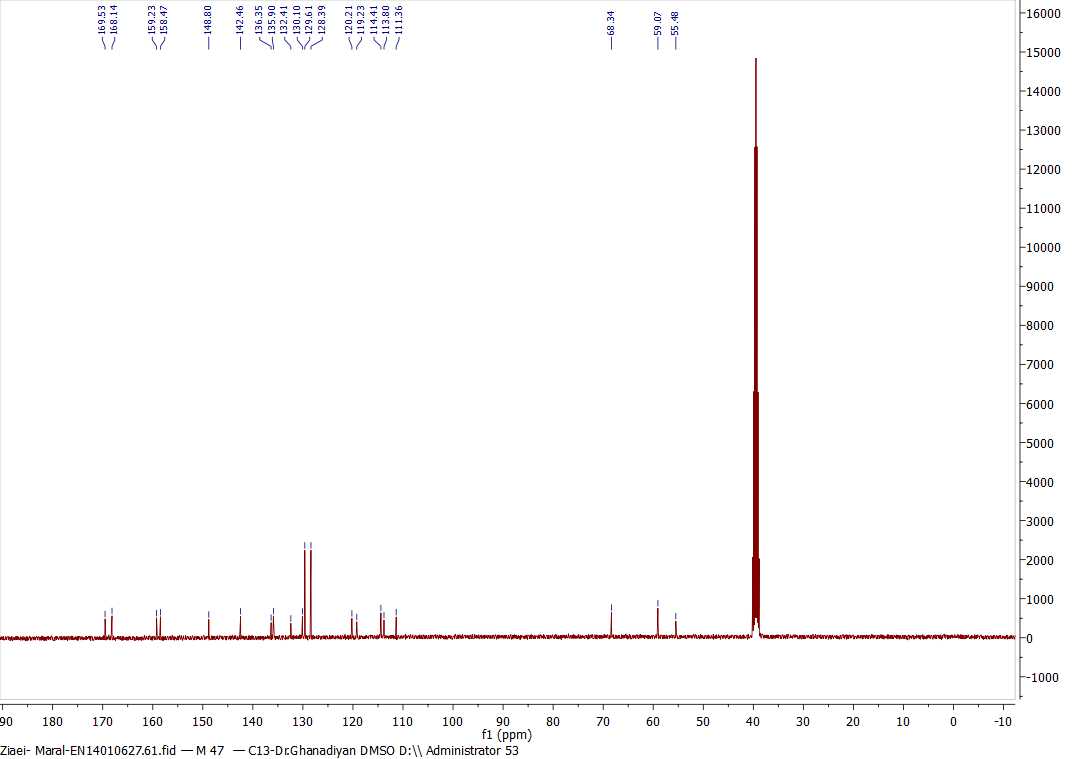
**

**Fig. S9. FT-IR, ^1^H NMR and ^13^C NMR spectrums of 2-amino-4-(3-((4-boromobenzyl)oxy)phenyl)-6-(hydroxymethyl)-8-oxo-4,8-dihydropyrano[3,2-b]pyran-3-carbonitrile (6i).**

**
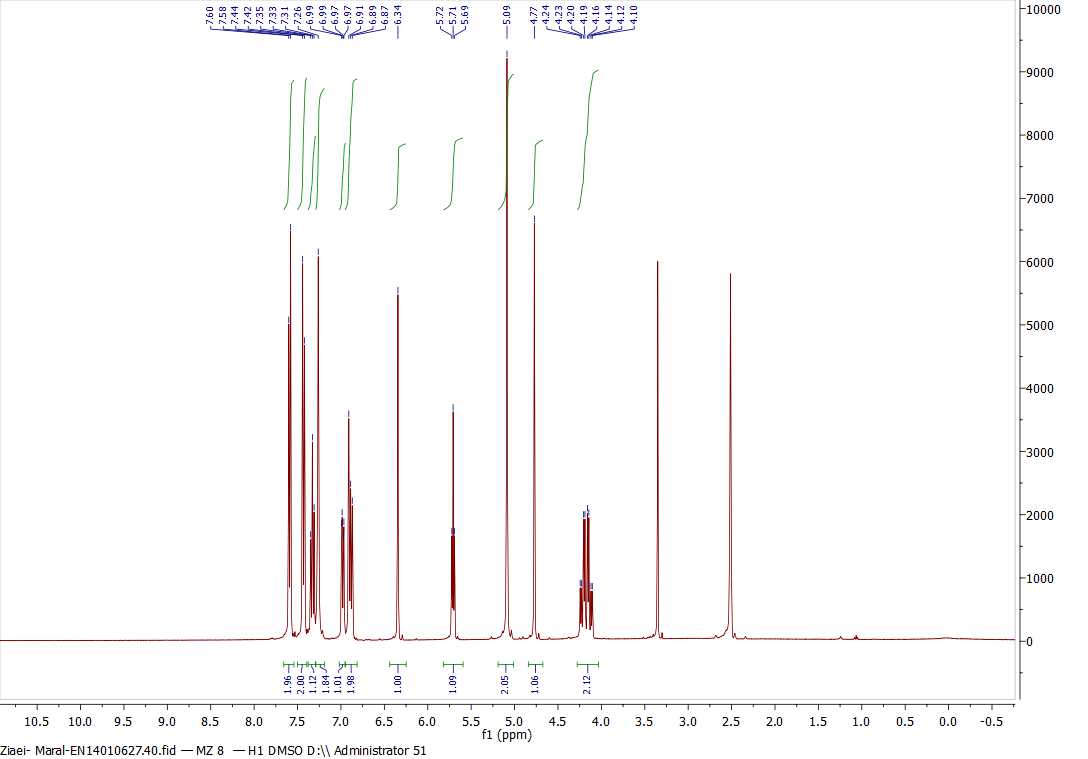
**

**
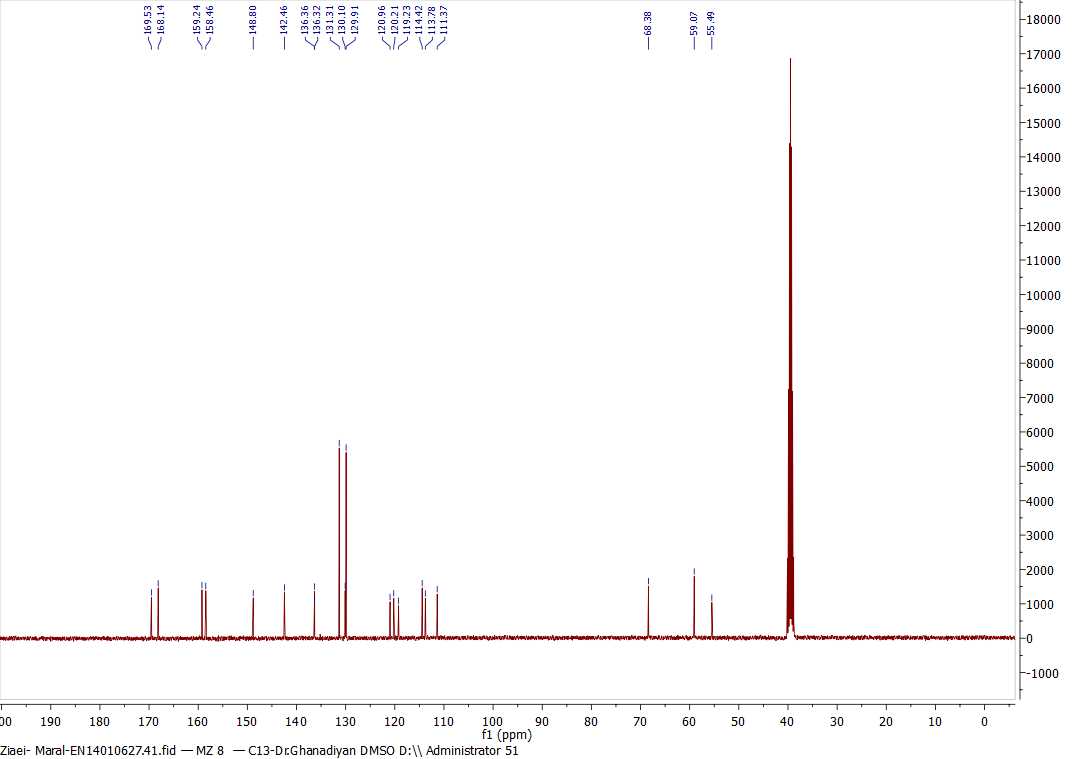
**

**Fig. S10. FT-IR, ^1^H NMR and ^13^C NMR spectrums of 2-amino-4-(3-((4-boromobenzyl)oxy)phenyl)-6-(hydroxymethyl)-8-oxo-4,8-dihydropyrano[3,2-b]pyran-3-carbonitrile (6j).**

**
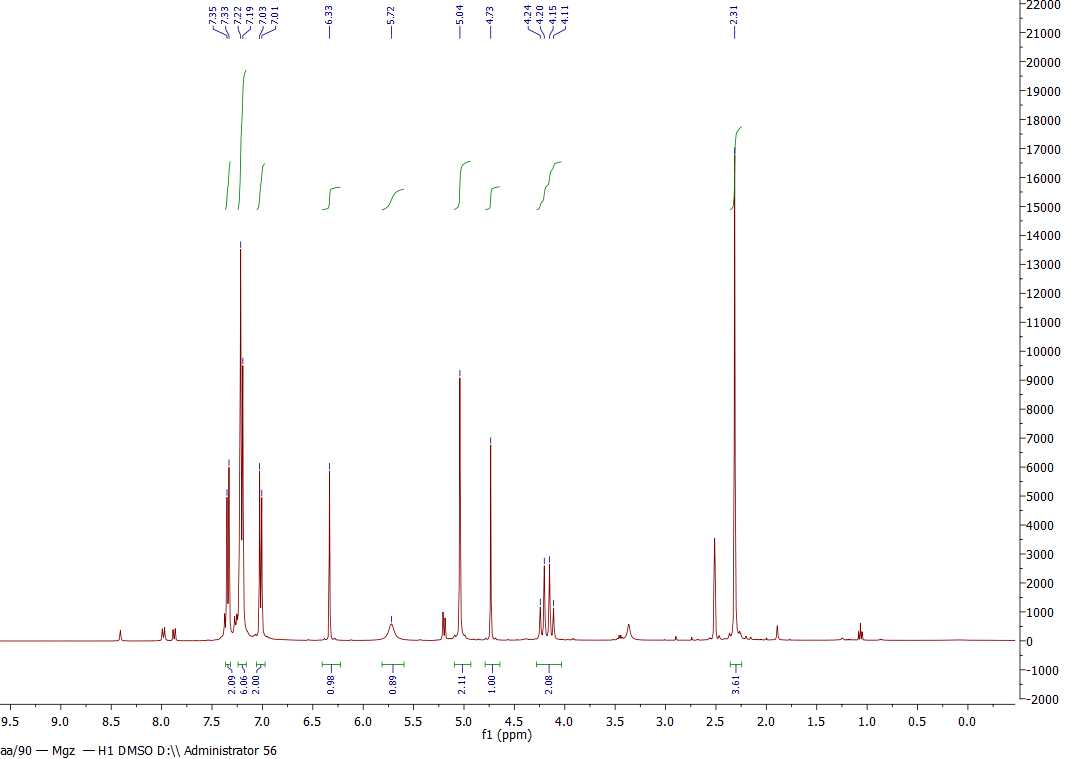
**


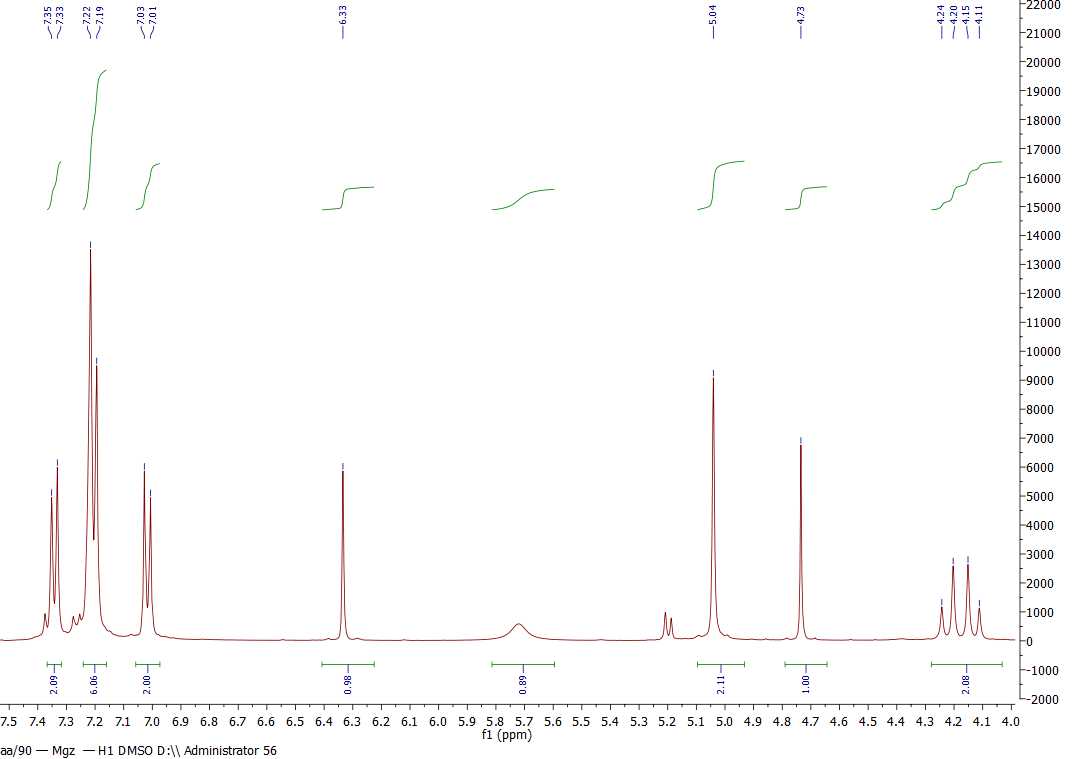


**
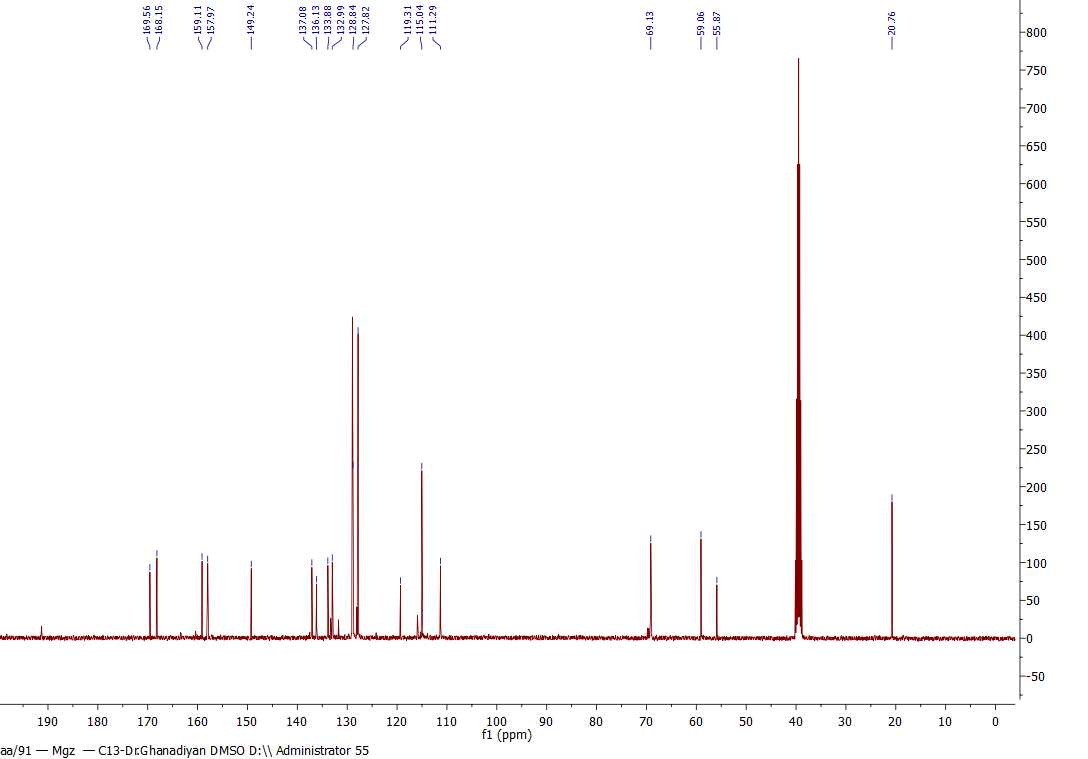
**

**Fig. S11. FT-IR, ^1^H NMR and ^13^C NMR spectrums of 2-amino-4-(4-(benzyloxy)-3-methoxyphenyl)-6-(hydroxymethyl)-8-oxo-4,8 dihydropyrano[3,2-b]pyran-3-carbonitrile (6k).**

**
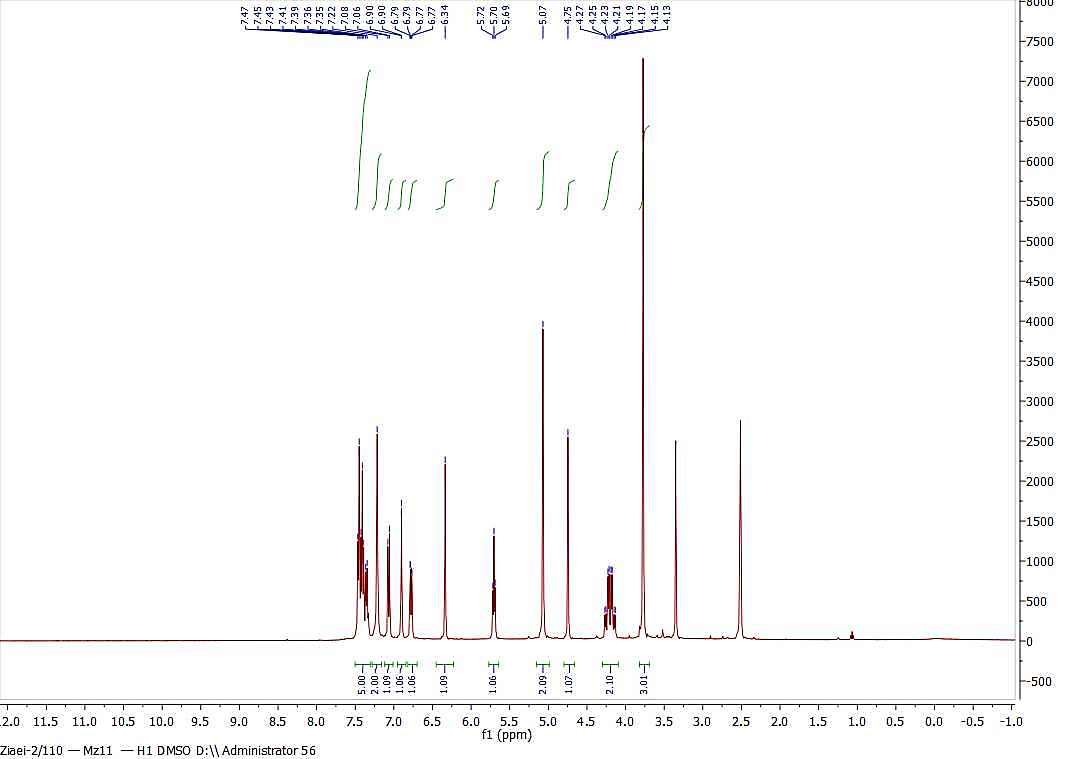
**

**
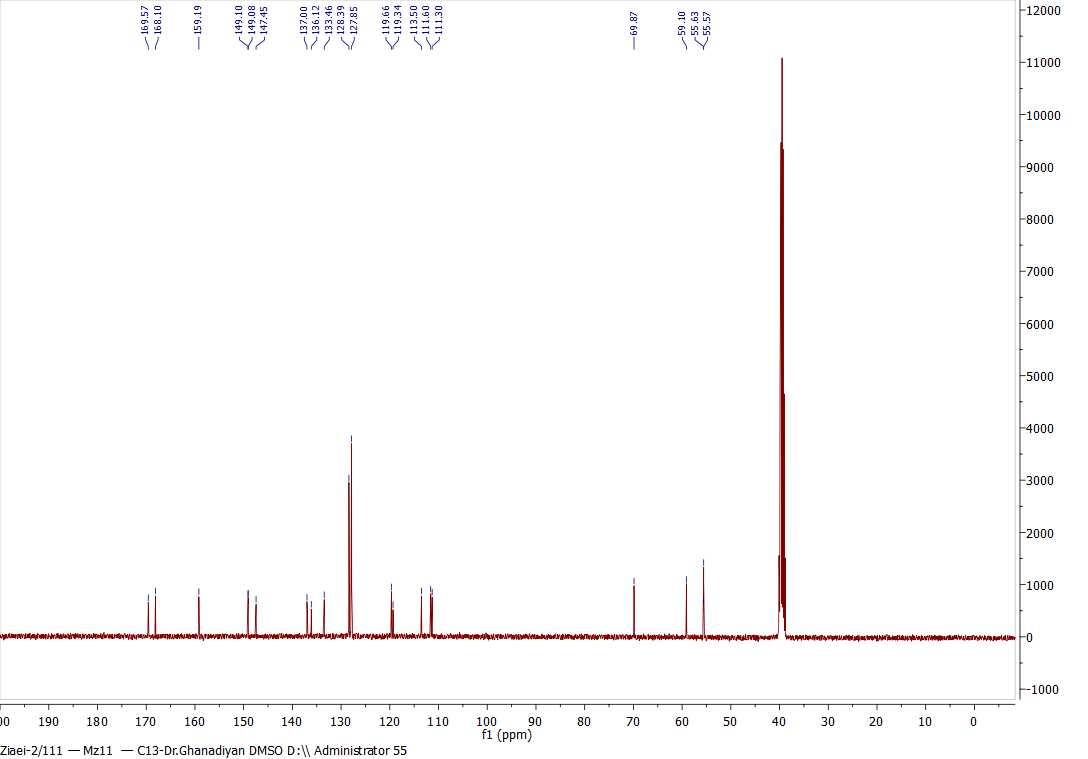
**

**Fig. S12. FT-IR, ^1^H NMR and ^13^C NMR spectrums of 2-amino-4-(4-((4-fluorobenzyl)oxy)-3-methoxyphenyl)-6-(hydroxymethyl)-8-oxo-4,8-dihydropyrano[3,2-b]pyran-3-carbonitrile (6l).**

**
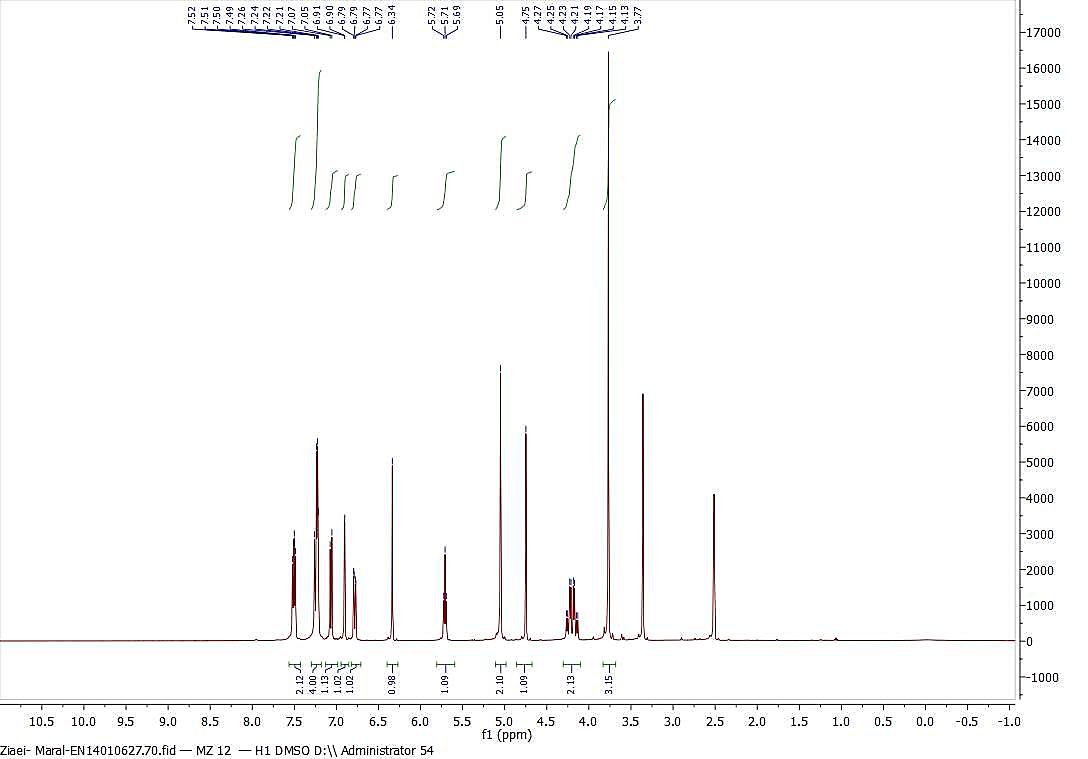
**

**
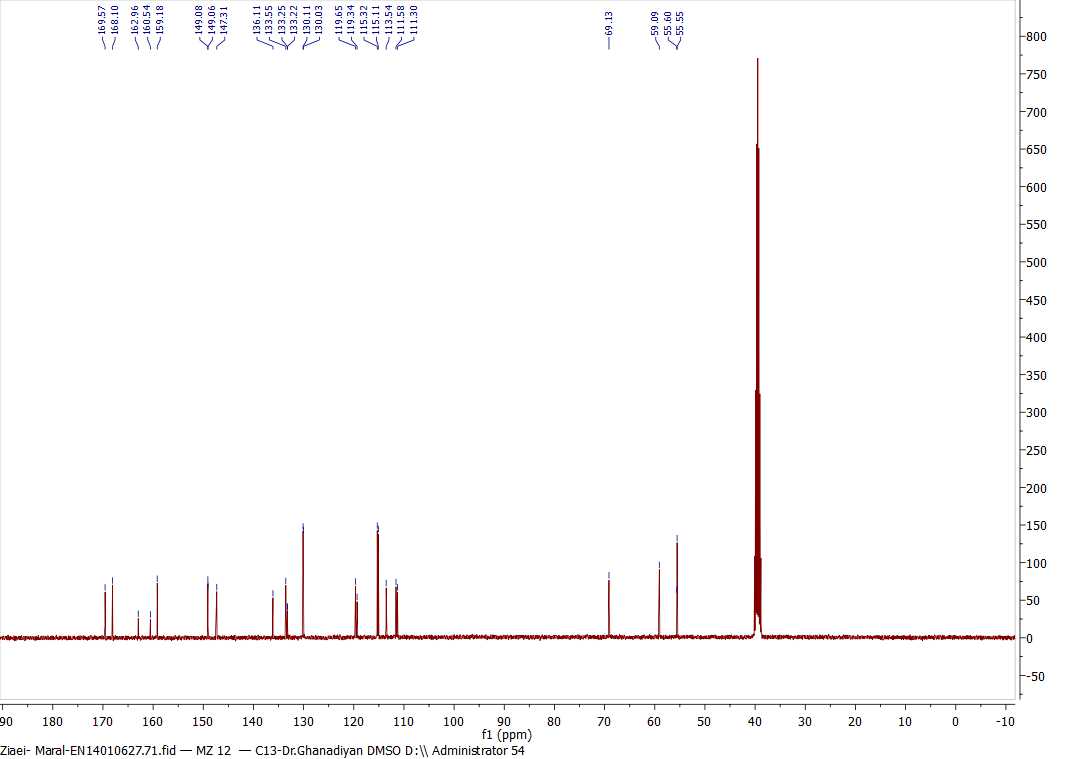
**

**Fig. S13. FT-IR, ^1^H NMR and ^13^C NMR spectrums of 2-amino-4-(4-((4-cholorobenzyl)oxy)-3-methoxyphenyl)-6-(hydroxymethyl)-8-oxo-4,8-dihydropyrano[3,2-b]pyran-3-carbonitrile (6m).**

**
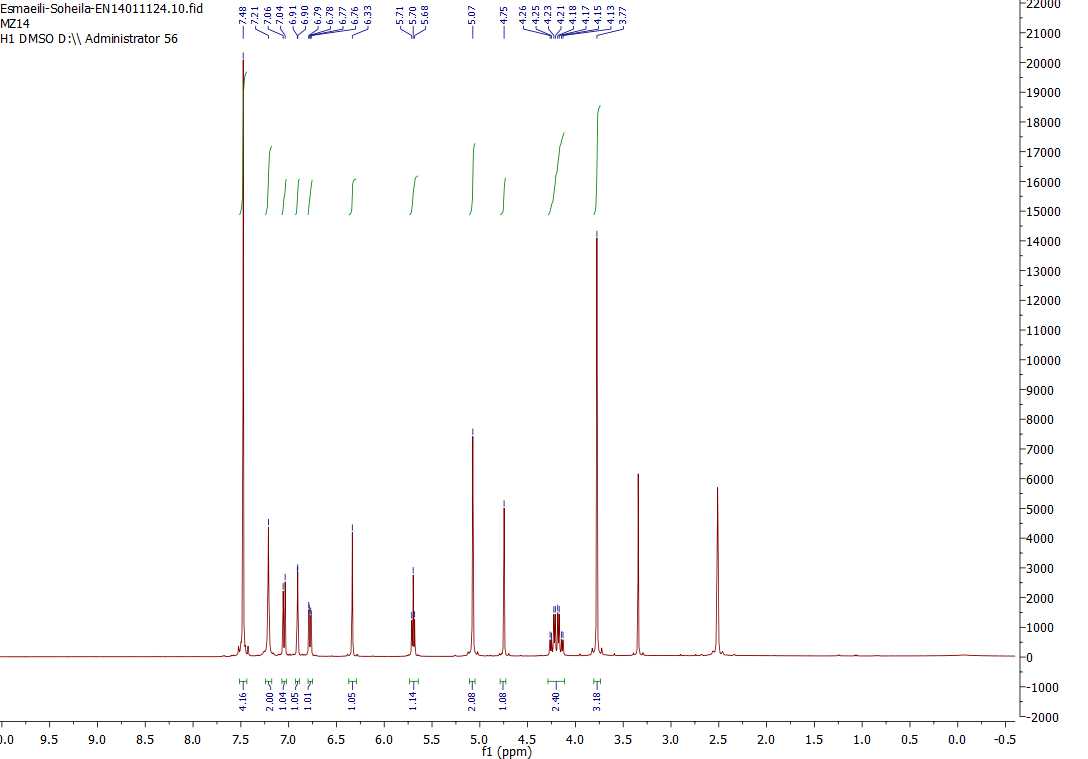
**

**
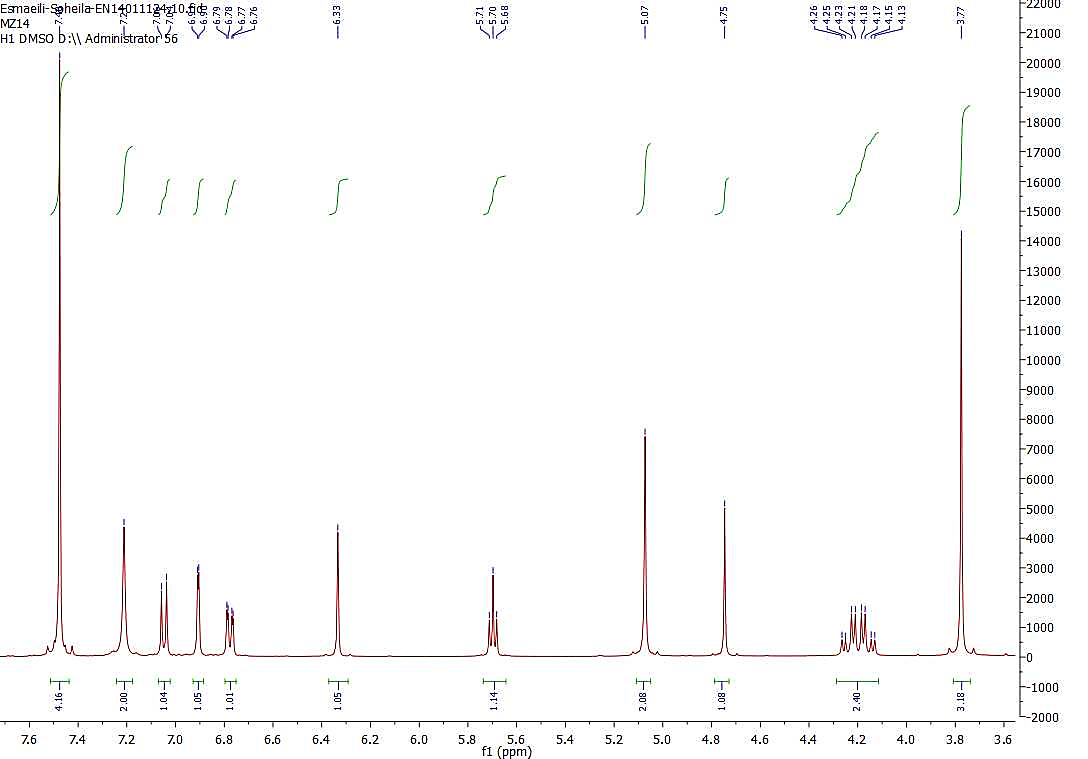
**


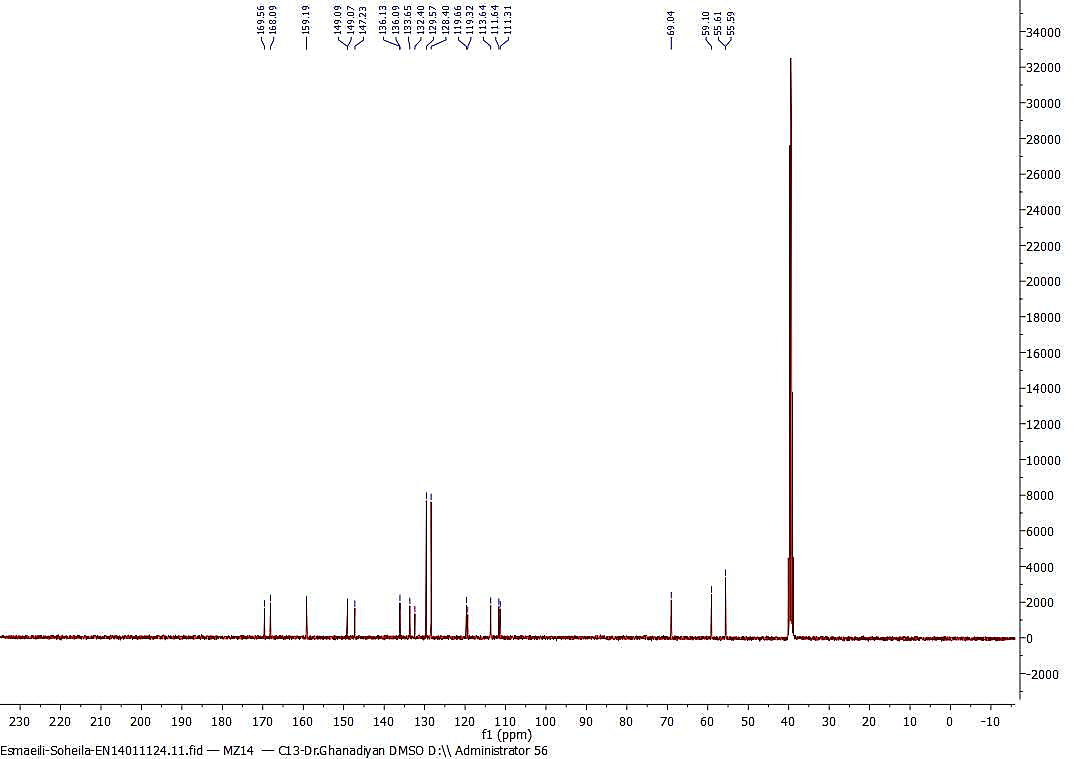


**Fig. S14. FT-IR, ^1^H NMR and ^13^C NMR spectrums of 2-amino-4-(4-((4-boromobenzyl)oxy)-3-methoxyphenyl)-6-(hydroxymethyl)-8-oxo-4,8-dihydropyrano[3,2-b]pyran-3-carbonitrile (6n).**

**
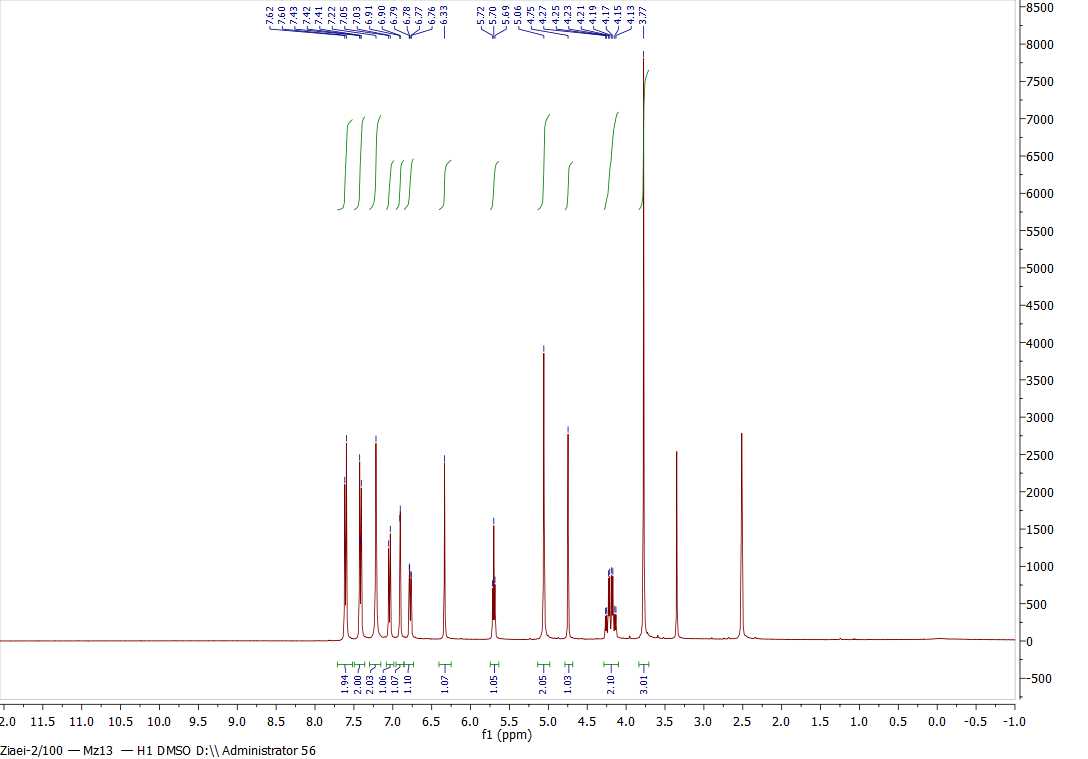
**


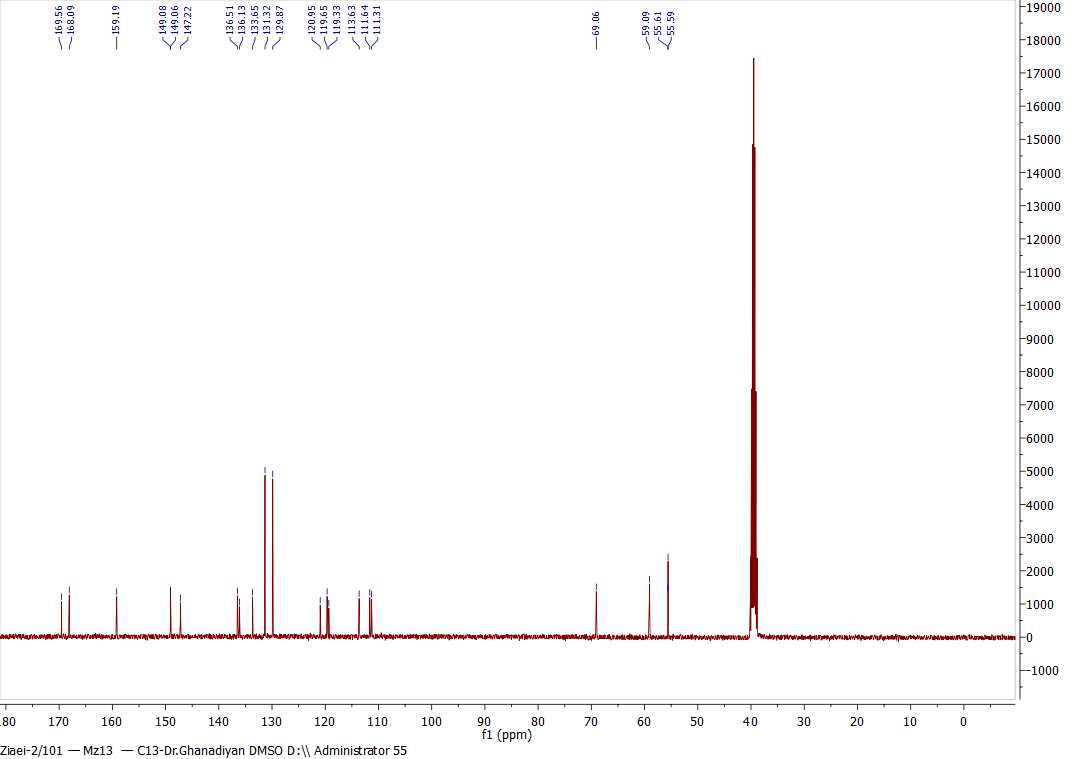


**Fig. S15. FT-IR, ^1^H NMR and ^13^C NMR spectrums of 2-amino-6-(hydroxymethyl)-4-(3-methoxy-4-((4-methylbenzyl)oxy)phenyl)-8-oxo-4,8 dihydropyrano[3,2-b]pyran-3-carbonitrile (6o).**


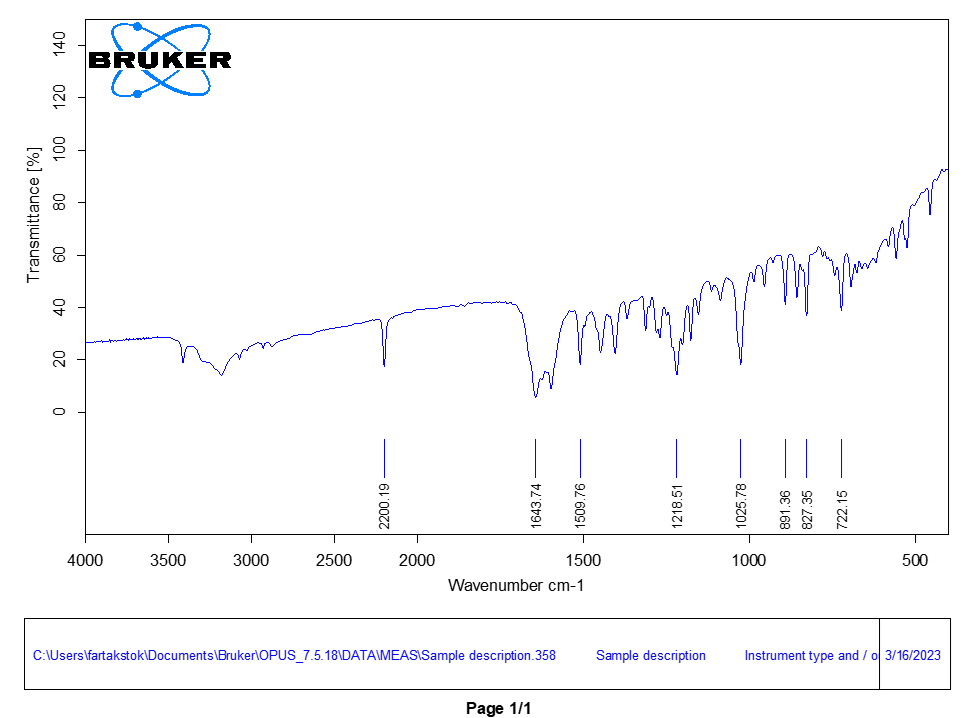


**
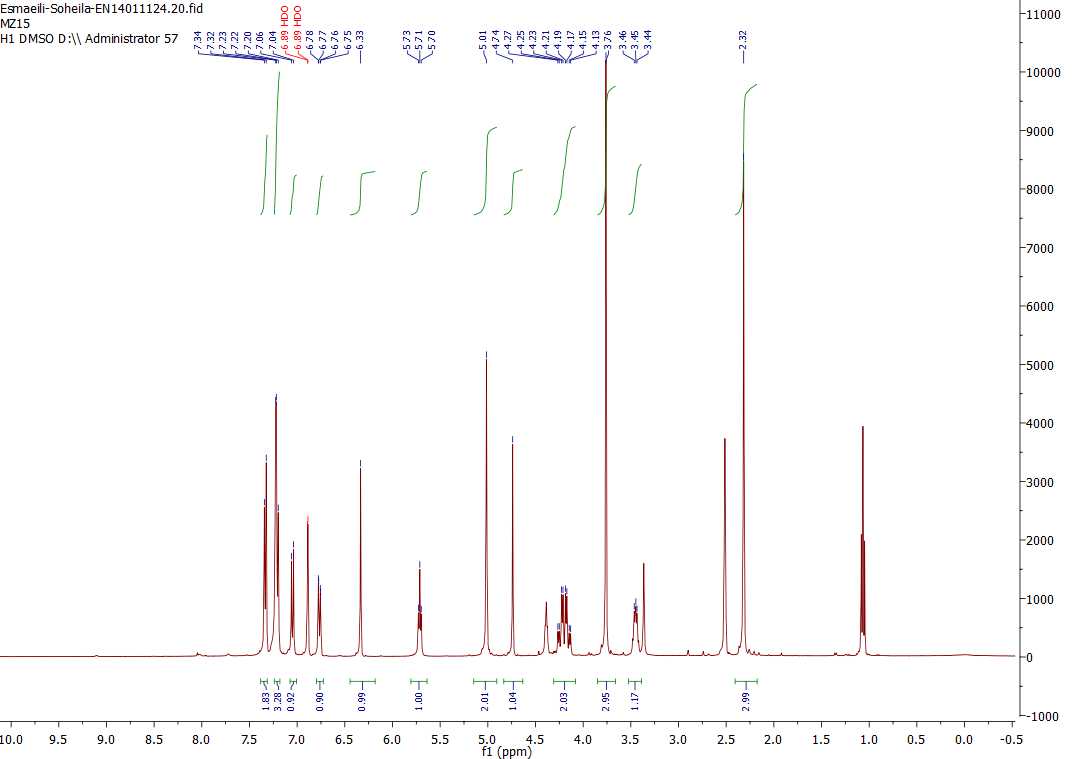
**

**
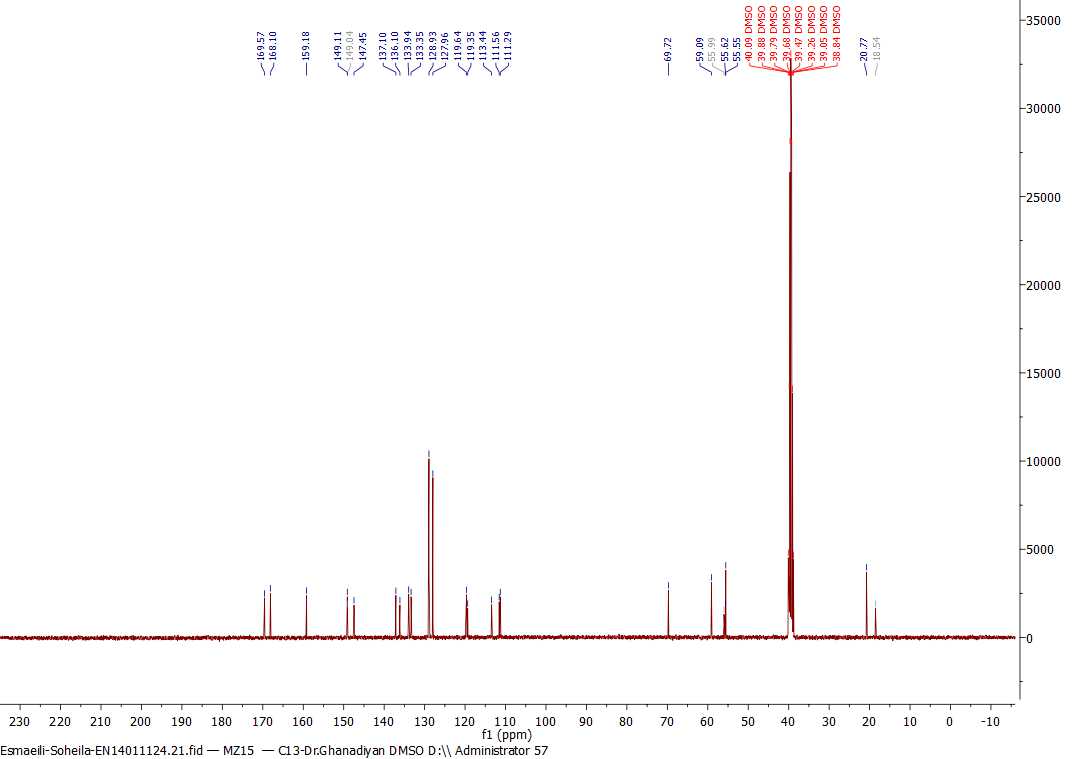
**
